# Critical roles of ARHGAP36 as a signal transduction mediator of Shh pathway in lateral motor columnar specification

**DOI:** 10.7554/eLife.46683

**Published:** 2019-07-15

**Authors:** Heejin Nam, Shin Jeon, Hyejin An, Jaeyoung Yoo, Hyo-Jong Lee, Soo-Kyung Lee, Seunghee Lee

**Affiliations:** 1 College of Pharmacy and Research Institute of Pharmaceutical SciencesSeoul National UniversitySeoulRepublic of Korea; 2 Neuroscience Section, Papé Family Pediatric Research Institute, Department of PediatricsOregon Health and Science UiversityPortlandUnited States; 3 College of Pharmacy and Inje Institute of Pharmaceutical Sciences and ResearchInje UniversityGyungnamRepublic of Korea; 4 Vollum InstituteOregon Health and Science UniversityPortlandUnited States; California Institute of TechnologyUnited States; The Francis Crick InstituteUnited Kingdom

**Keywords:** ARHGAP36, motor neuron, spinal cord, sonic hedgehog, Isl1, Lhx3, Chicken, Mouse

## Abstract

During spinal cord development, Sonic hedgehog (Shh), secreted from the floor plate, plays an important role in the production of motor neurons by patterning the ventral neural tube, which establishes MN progenitor identity. It remains unknown, however, if Shh signaling plays a role in generating columnar diversity of MNs that connect distinct target muscles. Here, we report that Shh, expressed in MNs, is essential for the formation of lateral motor column (LMC) neurons in vertebrate spinal cord. This novel activity of Shh is mediated by its downstream effector ARHGAP36, whose expression is directly induced by the MN-specific transcription factor complex Isl1-Lhx3. Furthermore, we found that AKT stimulates the Shh activity to induce LMC MNs through the stabilization of ARHGAP36 proteins. Taken together, our data reveal that Shh, secreted from MNs, plays a crucial role in generating MN diversity via a regulatory axis of Shh-AKT-ARHGAP36 in the developing mouse spinal cord.

## Introduction

Spinal motor neurons (MNs) innervating the limb are contained within the lateral motor columns (LMCs), which are produced specifically at brachial and lumbar levels of the spinal cord. LMC neurons are subsequently divided into two populations, lateral LMC (LMCl) neurons that innervate the dorsal part of the limb and medial LMC (LMCm) neurons that innervate the ventral part of the limb (Landmesser, 1978; Hollyday, 1980a; Hollyday, 1980b; Tosney and Landmesser, 1985b; Tosney and Landmesser, 1985a; Sockanathan et al., 2003; Ji et al., 2006). The molecular mechanism of how morphogenetic signaling molecules cooperate with transcription factors to define MN subtype specification has been extensively studied (Shirasaki and Pfaff, 2002; Lee and Pfaff, 2001). Retinoic acid (RA) is essential for the diversification of MN subtype and MN columnar organization. At brachial level, within the LMCs, RA is synthesized in subpopulations of MNs expressing the RA-synthesizing enzyme, RALDH2, and specifies migrating MN precursor cells into the LMCl neurons (Vermot et al., 2005; Sockanathan et al., 2003). However, it remains poorly understood whether other signaling molecules also contribute to the specification and/or maintenance of LMC and other MN columns.

Sonic hedgehog (Shh) signaling is a highly conserved pathway that is essential in directing cell proliferation and patterning during early embryogenesis (Ingham et al., 2011; Briscoe and Thérond, 2013). In spinal neuron development, Shh, released from the notochord and floor plate, leads to the generation of distinct classes of progenitor domains including MN progenitor (pMN) within the ventral side of spinal cord (Lee and Pfaff, 2001). In Shh-responsive cells, protein kinase A (PKA) plays crucial roles in fate specification and proliferation by modulating the activity of Shh signaling (Kotani, 2012; Asaoka, 2012). In the absence of Shh, PKA phosphorylates Gli transcription factors, promoting the production of repressor forms of Gli and thus repressing the Shh target gene expression, while Shh antagonizes this action of PKA (Pan et al., 2009). As both decreased and increased activity of PKA results in abnormal cell proliferation and cell fate specification, the basal level of PKA activity should be precisely controlled spatiotemporally to ensure its proper action (Kotani, 2012). PKA activation occurs upon binding of cyclic AMP (cAMP) to its regulatory subunits (PKAR), causing the release of its catalytic subunits (PKAC) (Taylor et al., 1990). Shh is able to induce phosphoinositide 3-kinase (PI3-kinase)-dependent AKT phosphorylation in cell lines such as LIGHT cells and HUVEC cells (Kanda et al., 2003; Riobó et al., 2006). Interestingly, PI3-kinase-dependent AKT activation further potentiates Shh signaling in the neuronal fate specification (Riobó et al., 2006). It was suggested that AKT might target proteins that modulate PKA kinase activity or the interaction between PKA and Gli (Riobó et al., 2006), but the identity of such proteins remain unknown.

During MN development, two LIM-homeodomain (LIM-HD) transcription factors, Islet-1 (Isl1) and LIM homeobox-3 (Lhx3) act as essential players in MN fate specification (Pfaff et al., 1996; Sharma et al., 1998; Tanabe et al., 1998; Thaler et al., 2002; Lee et al., 2013) by forming a hexameric complex, named the Isl1-Lhx3 complex, which consists of two Isl1:Lhx3 dimers and a nuclear LIM interactor (NLI) dimer (Lee et al., 2008; Lee et al., 2013). Genome-wide analyses of the Isl1-Lhx3 complex binding sites from chromatin immunoprecipitation-sequencing (ChIP-seq) datasets (Lee et al., 2013; Mazzoni et al., 2013) combined with MN transcriptome analyses (Lee et al., 2012; Mazzoni et al., 2013) identified many novel signaling pathways and regulators that are directly regulated by the Isl1-Lhx3 complex. Studies of individual target genes of the Isl1-Lhx3 complex in MN specification uncovered critical effector genes in MN specification such as genes involved in cholinergic neuronal identity determination (Cho et al., 2014), miR-218 as a downstream effector of Isl1-Lhx3 complex (Thiebes et al., 2015) and STAM1 as an endosomal sorting machinery necessary for ventral motor axon projection (Nam and Lee, 2016).

ARHGAP36, a putative Rho GTPase-activating protein, was identified from a genome-scale cDNA overexpression screen as a positive regulator of the Shh pathway (Rack et al., 2014). Overexpression of ARHGAP36 recapitulates Shh signaling transduction and these effects are independent of Smo and require kinesin family member 3a (Kif3a) and intraflagellar transport protein 88 (Ift88) (Rack et al., 2014). Recently, ARHGAP36 has emerged as a potent antagonist of PKA signaling. ARHGAP36 interacts with PKAC and inhibits PKAC catalytic activity as a PKA pseudosubstrate inhibitor (Eccles et al., 2016). It also targets PKAC for ubiquitin-dependent proteolysis by the endosomal sorting complex required for transport (ESCRT) pathway (Eccles et al., 2016). Moreover, ARHGAP36 interaction with Patched1 leads to the removal of centrosomal ARHGAP36 with ciliary Patched1 and the accumulation of centrosomal PKA that phosphorylates Inversin and this Patched1-ARHGAP36-PKA-Inversin axis determines the ciliary translocation of Smoothened and consequent hedgehog pathway activation (Zhang et al., 2019). Although recent studies in cultured cells suggest that ARHGAP36 regulates Shh activity through inhibiting PKA kinase activity, it remains unexplored whether ARHGAP36 functions as a key modulator of Shh signaling pathway in vivo.

Here we report that Shh expression is induced in postmitotic MNs at brachial and lumbar levels but not at thoracic level at later stages of development when motor columnar identities are established. Shh is required for proper LMC formation as the knock-down of *Shh* in the developing chick spinal cord and the deletion of *Shh* in the developing MNs of mouse embryos result in reduction of FoxP1^+^ LMC. We further show that ARHGAP36 is a critical MN-enriched Shh transduction component and *Arhgap36* is a direct target gene of Isl1-Lhx3 complex during MN generation. The action of ARHGAP36 is to promote Gli-dependent transactivation partly through inhibition of PKA activity. Moreover, AKT, known as an inhibitor of PKA activity, interacts with ARHGAP36 and stabilizes ARHGAP36 protein, which further enforces suppression of PKA activity by ARHGAP36. Consistently, blocking AKT activity reduces the protein level of ARHGAP36 and hinders the efficiency of in vitro MN differentiation from mouse ESCs. Consistently, deletion of *Arhgap36* gene in mouse results in defects in LMC formation at brachial level, which might be caused by dysregulation of Shh signaling pathway. Our results define a new regulatory axis of Shh-AKT-ARHGAP36-PKA in LMC specification, in which ARHGAP36 functions as an essential effector of Shh and AKT in repressing PKA activity.

## Results

### Shh is expressed in LMC neurons in developing mouse and chick spinal cord

**Figure 1.**
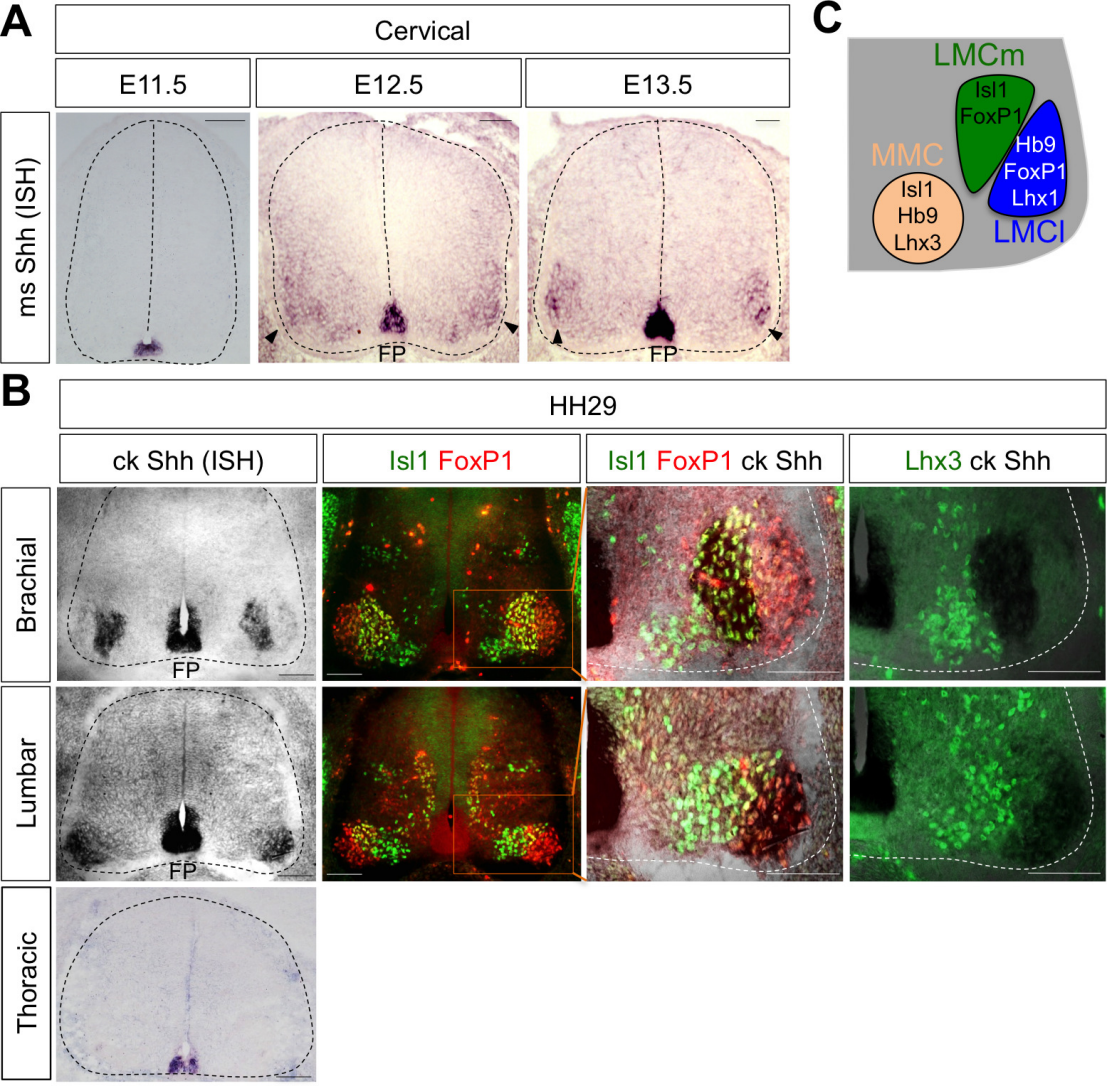
Shh is expressed in LMC neurons in developing mouse and chick spinal cord. (**A**) ISH analysis of mouse spinal cord (dotted outline) showed the expression of Shh in FP and LMC region (arrowhead) at later stages when motor columns are specified. (**B**) ISH analysis of chick Shh combining with IHC of Isl1/FoxP1 and Lhx3 in chick spinal cord. At HH St.29, Shh is mainly detected in LMCm (Isl1^+^/FoxP1^+^) neurons at brachial levels and LMCl (Isl1^-^/FoxP1^+^) neurons at lumbar levels but not in motor neurons at thoracic levels of chick spinal cord (dotted outline). Scale bars: 100 μm. (**C**) Schematic drawing shows the LMCm, LMCl, and MMC motor columns in the ventral spinal cord with representative markers.

While searching for extrinsic signaling molecules for LMC specification other than RA, we found that Shh shows an interesting expression pattern in ventro-lateral region of spinal cord where LMC neurons are located. At earlier stages, Shh is mainly detected in the notochord (NC) and floor plate (FP) of the mouse and chick embryos (Bitgood and McMahon, 1995; Oppenheim et al., 1999; Martí and Bovolenta, 2002) (Figure 1A). When MNs begin to be segregated into distinct motor columns, Shh is also detected in the LMC region at brachial and lumbar level but not at the thoracic level in chick (Bitgood and McMahon, 1995; Oppenheim et al., 1999; Martí and Bovolenta, 2002) and mouse embryos (Figure 1A and B). To provide the detailed expression of Shh, we performed in situ hybridization (ISH) for chick Shh and immunohistochemistry (IHC) for well-defined markers for motor columns in chick embryos (HH St.29). Our analyses showed that Shh is expressed in LMCm (Isl1^+^/FoxP1^+^) but not LMCl (Isl1^-^/FoxP1^+^) at brachial level, and interestingly, it is expressed in LMCl (Isl1^-^/FoxP1^+^) at lumbar level. Shh expression is clearly excluded from MMC (Lhx3^+^ MNs) at all axial levels, as demonstrated by converging analyses of Lhx3 and Shh (Figure 1B and C). The expression of Shh in motor neurons of mouse embryo is much lower than that of in chick embryo and it is rather restricted to LMCl region at different developmental stages examined so far (Figure 1A). As we examined the comparable developmental stages in mouse and chick embryos, the sensitivity differences in ISH outcomes may reflect species differences. Nonetheless, our analyses in both mouse and chick embryos demonstrate the expression of Shh in postmitotic motor neurons.

### Shh, expressed in LMC neurons, is necessary for LMC specification in developing chick spinal cord

**Figure 2.**
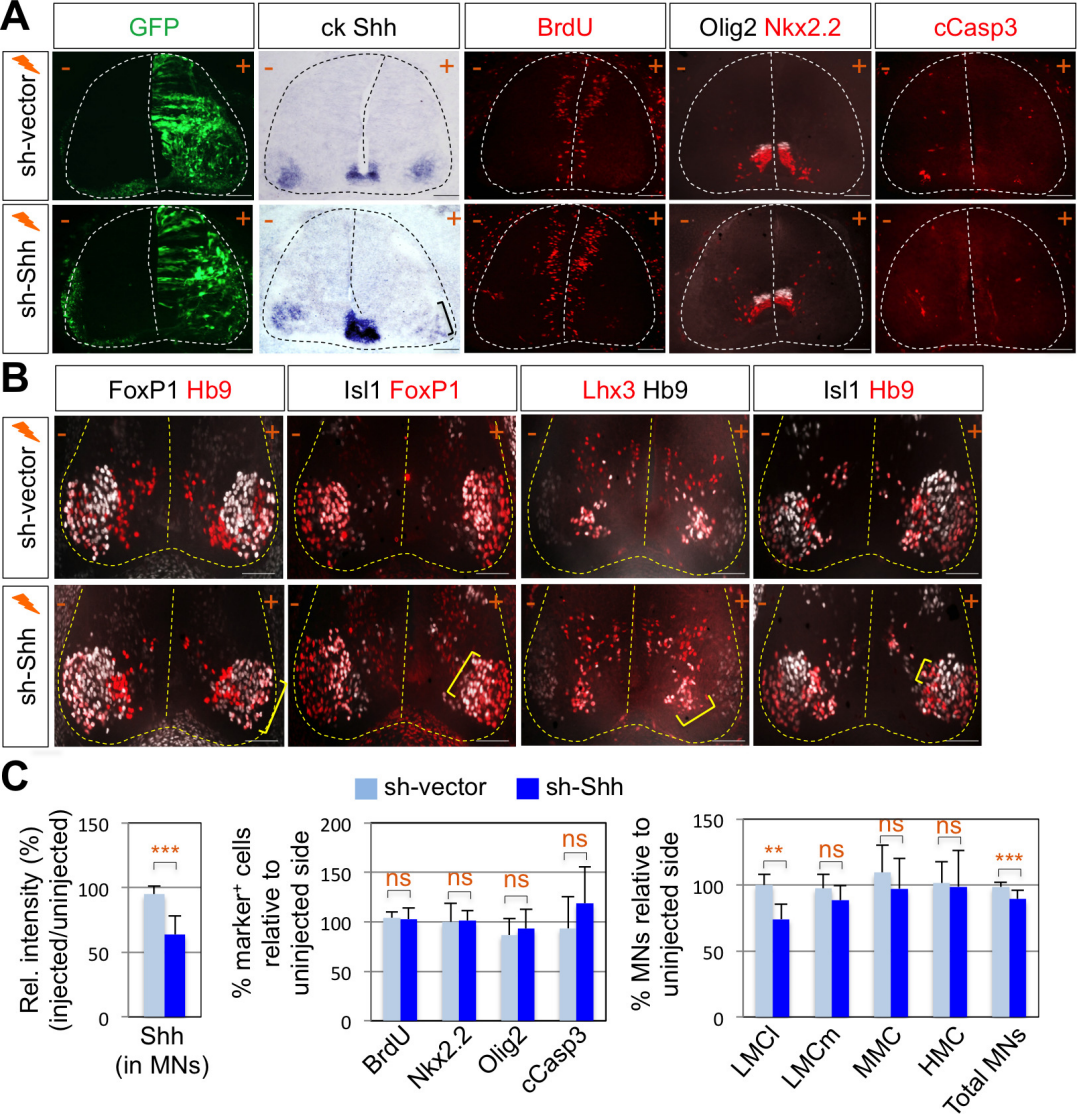
Shh signaling is required for LMC specification in chick spinal cord. (**A**) IHC analyses in chick spinal cords electroporated with sh-Shh and sh-vector construct. ISH analysis showed the reduced expression of chick Shh in sh-Shh electroporated chick embryo but not in control sh-vector injected chick embryo. Knock-down of chick Shh did not affect the proliferation (BrdU) or survival (cCasp3) of neural progenitor cells and ventral neural patterning (Olig2 and Nkx2.2). (**B**) Knock-down of chick Shh reduced the number of LMCl (Hb9^+^/FoxP1^+^) neurons but had no effect on other motor columns such as LMCm, MMC, and HMC and consequently reduced the number of total MNs compared to the uninjected control side. +, electroporated side; -, non-electroporated control side. (**C**) Quantification of the relative intensity of Shh ISH signal in motor neurons, % marker^+^ (BrdU, Nkx2.2, Olig2, and cCasp3) cells relative to uninjected side and % motor columns relative to uninjected side of the spinal cord. Each set of chick electroporation experiments in this figure was repeated independently at least three times with 6 to 10 embryos. Embryos were harvested 4 days post electroporation (dpe). Data are mean ± s.d. **p<0.001, ***p<0.0001; ns, non-significant (Student’s t-test). n = 6 ~ 15 independent images per each sample. Scale bars: 100 μm. 10.7554/eLife.46683.004Figure 2—source data 1.Source data for Figure 2C.

To test whether Shh contributes to motor columnar fate determination, we generated a short-hairpin RNA (shRNA) construct that targets chick Shh. We misexpressed shRNA-Shh or shRNA-vector control in neural progenitors of the developing spinal cord by using *in ovo* electroporation and harvested embryos 4 days post electroporation when motor columns are established. *In ovo* electroporation of shRNA vector resulted in the expression of GFP throughout the electroporated spinal cord (Figure 2A). First, we confirmed the specific reduction of *Shh* expression in LMC region but not in the floor plate of the spinal cord (Figure 2A and C). In our electroporation condition, the electroporation efficiency of the floor plate cells is generally very low. Thus, we expected that Shh knock-down would be mostly effective in motor neurons but not in the floor plate, allowing us to focus on analyzing the effect of Shh loss-of-function (LOF) on motor columnar specification without significant changes in progenitor proliferation or early patterning of the neural tube. Furthermore, we analyzed the chick embryos that do not express GFP in the floor plate, which was further confirmed by ISH analyses of Shh expression (Figure 2A and C). Our analyses including careful quantification indeed revealed that there’s no significant change in proliferation (BrdU^+^ cells), survival (cCasp3^+^ cells) of neural progenitors or ventral neural patterning (Olig2^+^ and Nkx2.2^+^ cells) (Figure 2A and C). These rigorous analyses exclude the possibility that any observed phenotypes are caused by the effect of Shh deletion in the progenitors. Newborn motor neurons express Isl1, Lhx3 and Hb9. As the motor neurons are segregated into different motor columns, MMC neurons keep on expressing Isl1, Lhx3 and Hb9 while LMC neurons lose Lhx3 expression and gain FoxP1 expression. Thus, FoxP1 marks LMC neurons including both LMCm (Isl1^+^/FoxP1^+^) and LMCl (Hb9^+^/FoxP1^+^) neurons, whereas Isl1 and Hb9 label both MMC and hypaxial motor column (HMC) neurons (Figures 1C and 5E). The number of LMCl (Hb9^+^/FoxP1^+^) neurons of the sh-Shh injected embryos showed approximately 26% reduction compared to the uninjected control side (Figure 2B and C). However there was no effect on other motor columns, and consequently the number of total MNs was reduced by knock-down of Shh (Figure 2B and C), suggesting that Shh plays an essential role in specifying the FoxP1^+^ LMC neuronal identity.

### Shh is also required for LMC formation in developing mouse spinal cord

**Figure 3.**
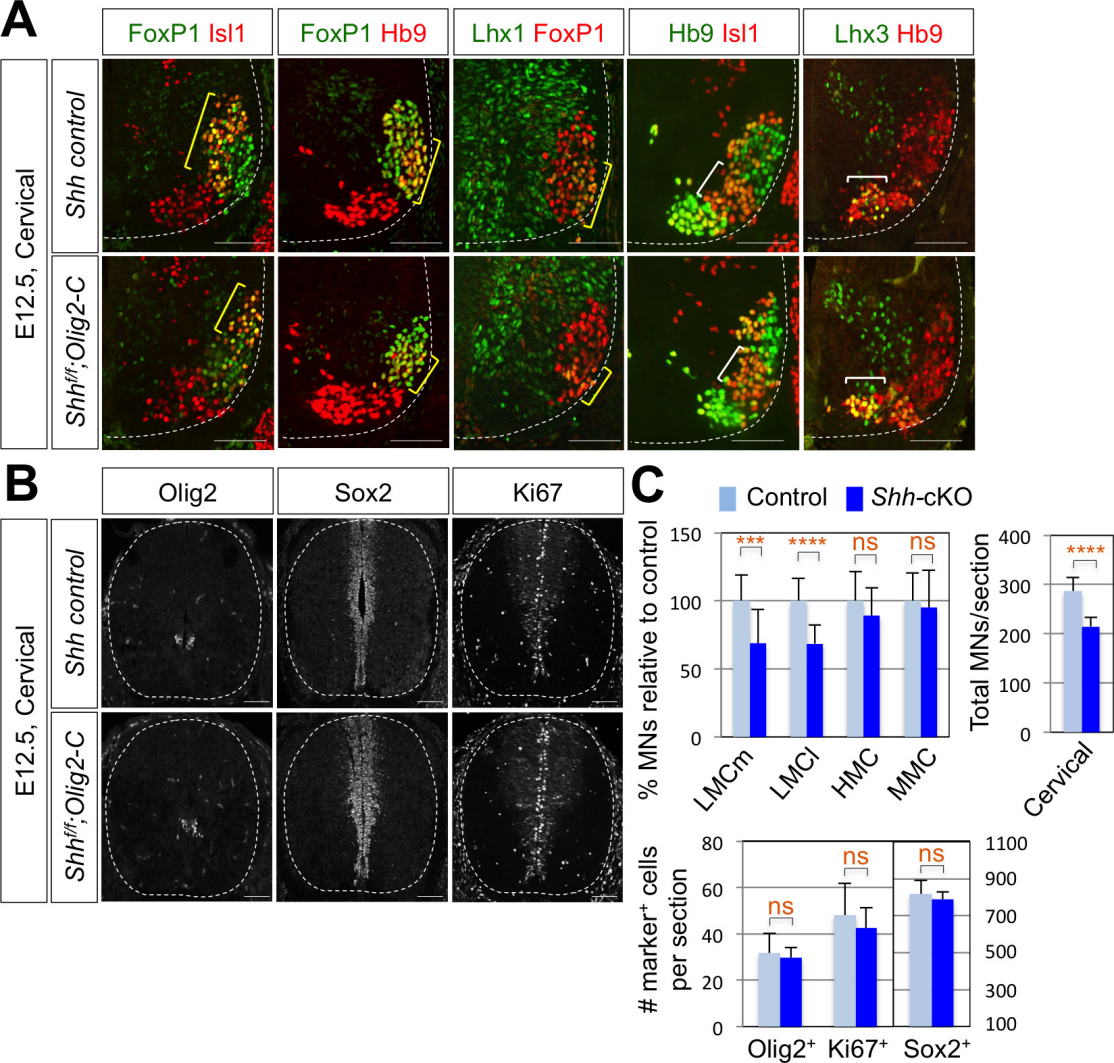
Shh is required for LMC formation in developing mouse spinal cord. (**A**) IHC analyses of E12.5 *Shh-*cKO (*Shh^f/f^;*Olig2-Cre) mutant embryos (n = 4) (lower panel) and control littermates (n = 4) (upper panel). The cervical level of ventral spinal cord is shown. LMCm (Isl1^+^/FoxP1^+^) neurons and LMCl (Hb9^+^/FoxP1^+^ or Lhx1^+^/FoxP1^+^) neurons (yellow bracket) in *Shh* conditional knock-out (*Shh*-cKO) were significantly reduced. On the other hand, the number of MMC (Hb9^+^/Lhx3^+^) and HMC (Hb9^+^/Isl1^+^) neurons did not change (white bracket). (**B**) IHC analyses of Olig2, Sox2, and Ki67 in E12.5 *Shh-*cKO mutant embryo and control littermates (cervical level). No significant difference in the expression of Sox2, Olig2 and Ki67 within the spinal cord. Scale bars: 100 μm. (**C**) Quantification of the number of LMCm (Isl1^+^/FoxP1^+^), LMCl (Hb9^+^/FoxP1^+^ or Lhx1^+^/FoxP1^+^), MMC (Hb9^+^/Lhx3^+^) and HMC (Hb9^+^/Isl1^+^) neurons, Olig2^+^, Sox2^+^, Ki67^+^ cells and total MNs at cervical level in E12.5 mouse embryonic spinal cord. Data are mean ± s.d. ***p<0.0001, ****p<0.00001; ns, non-significant (Student’s t-test). n = 5 ~ 28 independent images per each sample. 10.7554/eLife.46683.008Figure 3—source data 1.Source data for Figure 3C.

To further support the function of Shh in LMC formation, we tried to delete *Shh* gene in mouse MNs by crossing *Shh*^f/f^ mice with MN specific Cre recombinase expressing mice. Hb9-Cre turned out to be problematic for our experiments, because Hb9 is expressed (therefore Hb9-Cre is active) in the notochord, which secretes Shh required for the neural tube development (Harrison et al., 1999). Isl1-Cre, whose Cre expression occurs as motor neurons emerge from progenitors, might lead to severe defects in the limb development as Isl1-Cre inactivates Shh in the developing limb (Harfe et al., 2004; Yang et al., 2006; Itou et al., 2012), which can complicate our analyses of LMC motor neuron development. Finally, Olig2-Cre mice in which the Cre recombinase is active in MN progenitors, but not in the floor plate cells (Dessaud et al., 2007; Sagner et al., 2018), was used to inactivate Shh in postmitotic MNs. Consistent with the results of reduced LMCs in chick spinal cord by knock-down of Shh, there was ~30% reduction of LMCm (Isl1^+^/FoxP1^+^) neurons and LMCl (Hb9^+^/FoxP1^+^ or Lhx1^+^/FoxP1^+^) neurons in *Shh* conditional knock-out (*Shh*-cKO) embryos compared to that of WT control littermate embryos at embryonic day (E) 12.5 (Figure 3A and C). But neither MMC (Hb9^+^/Lhx3^+^) neurons nor HMC (Hb9^+^/Isl1^+^) neurons were affected (Figure 3A and C), which resulted in reduction of total number of MNs in *Shh*-cKO compared to control littermates. These results suggest that Shh plays an essential role in specifying the FoxP1^+^ LMC neuronal identity but not MMC (Hb9^+^/Lhx3^+^) or HMC (Hb9^+^/Isl1^+^) neuronal identity in developing mouse spinal cord.

**Figure 3—figure supplement 1.**
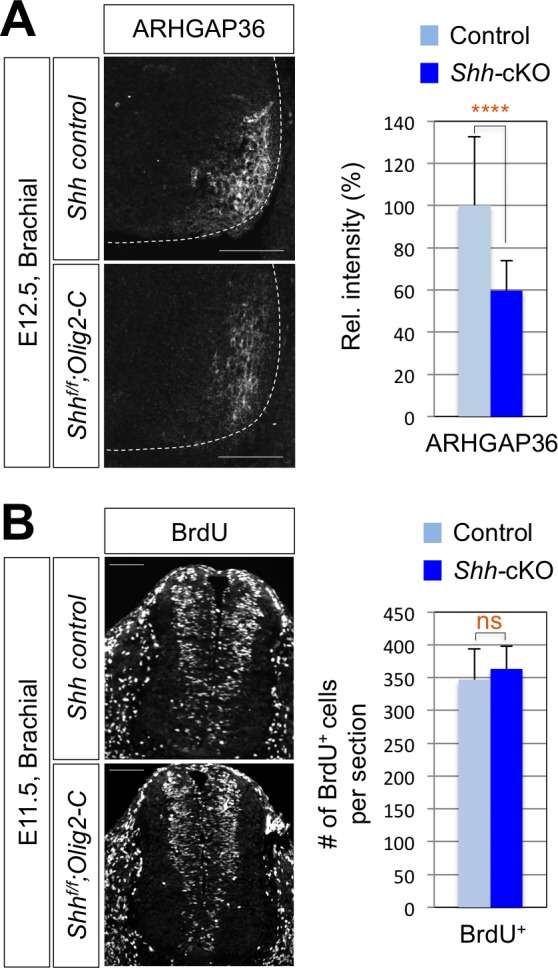
Reduced expression of ARHGAP3 does not affect progenitor cell proliferation in *Shh*-cKO mouse spinal cord. (**A**) IHC analysis for ARHGAP36 in E12.5 *Shh*-cKO (n = 4) and littermate control (n = 4) embryos showing the reduced expression of ARHGAP36 in ventro-lateral region of the spinal cord at cervical level. (**B**) IHC analysis for BrdU incorporation in E11.5 *Shh*-cKO (n = 4) and littermate control (n = 5) embryos showing no defect in proliferation of progenitor cells within the spinal cord. Scale bars: 100 μm. Data are mean ± s.d. ****p<0.00001; ns, non-significant (Student's t-test). n = 14 ~ 32 independent images per each sample. 10.7554/eLife.46683.007Figure 3—figure supplement 1—source data 1.Source data for Figure 3—figure supplement 1.

To test whether the reduced number of FoxP1^+^ cell was resulted from the defects in the proliferation of the neural stem cells, in particular the MN progenitors, we examined Ki67^+^ cells, BrdU incorporation, and expression patterns of Sox2 that labels the proliferating progenitor cells in the ventricular zone and Olig2, a marker of MN progenitors (pMN) (Figure 3B and C and Figure 3—figure supplement 1B). There was no significant difference in the expression of Ki67, BrdU, Sox2 and Olig2 within the spinal cord of *Shh*-cKO mutants compared with control embryos (Figure 3C), suggesting that the specific deletion of Shh in MNs does not perturb the proliferation of neural stem cells and the overall dorsal-ventral patterning of the spinal cord.

### *Arhgap36* is identified as a direct target gene of the Isl1-Lhx3 complex

**Figure 4.**
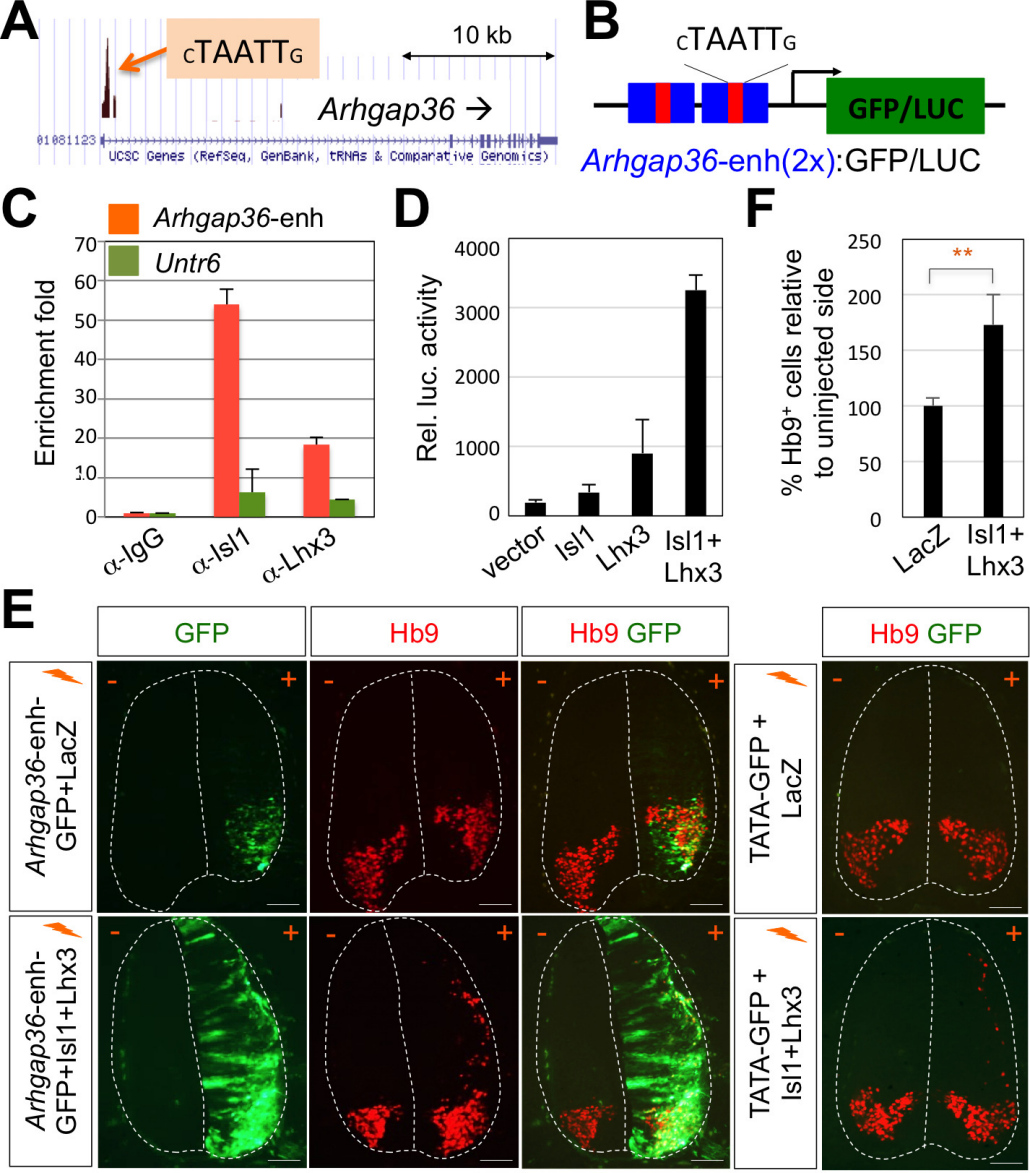
ChIP-seq peaks for the Isl1-Lhx3 complex in *Arhgap36* and their in vivo recruitment of the Isl1-Lhx3 complex. (**A**) Isl1-Lhx3 complex binding sites in *Arhgap36*. The peak has HxRE motif. (**B**) A schematic representation of reporter constructs linked to two copies of *Arhgap36*-enhancer genomic DNA fragment. (**C**) Both Isl1 and Lhx3 were recruited to Isl1-Lhx3-bound ChIP-seq peak in *Arhgap36* gene. ChIP was performed with anti-IgG antibody (control), anti-Isl1 and anti-Lhx3 antibodies using E12.5 mouse embryonic spinal cord extracts. Quantitative PCR amplification of the binding region of *Arhgap36* and negative control region, *Untr6*. ChIP experiments were repeated independently twice. Data are presented as the mean of duplicate values and error bars represent standard deviation. (**D**) Luciferase assay for a reporter directed by two copies of *Arhgap36-enhancer*. Transfections were repeated independently at least three times. Data are presented as the mean of triplicate values and error bars represent standard deviation. (**E**) *In ovo* electroporation of LacZ (to measure electroporation efficiency) and a GFP reporter directed by two copies of *Arhgap36*-enhancer without or with co-expression of Isl1 and Lhx3. TATA-GFP vector with no HxRE was used as a negative control and this reporter was not activated even when Isl1 +Lhx3 expression induces ectopic MNs in dorsal spinal cord. Each set of DNA was injected and electroporated in chick neural tube and embryos (n = 5 ~ 10) were harvested 3 days post electroporation (three dpe). Hb9 staining labels endogenous and ectopically induced motor neurons in the spinal cord. +, electroporated side, –, non-electroporated side. White dotted lines indicate the outline of the spinal cord. Experiments were repeated independently at least three times. Scale bars: 100 μm. (**F**) Quantification of the number of Hb9^+^ cells relative to uninjected side of the spinal cord. Data are mean ± s.d. **p<0.001 (Student’s t-test). n = 5 ~ 8 independent images per each sample. 10.7554/eLife.46683.010Figure 4—source data 1.Source data for Figure 4C. 10.7554/eLife.46683.011Figure 4—source data 2.Source data for Figure 4D. 10.7554/eLife.46683.012Figure 4—source data 3.Source data for Figure 4F.

Given this novel action of Shh in MNs is distinct from the established role of Shh pathway in neural progenitors for patterning the ventral neural tube, we considered the possibility that MN-specific downstream effector of Shh mediates the Shh activity in driving LMC formation. To identify the candidate effector genes, we searched for target genes of the Isl1-Lhx3 by analyzing the Isl1-Lhx3-bound genomic loci mapped by ChIP-seq analyses (Mazzoni et al., 2013; Lee et al., 2013). Among several putative target genes of the Isl1-Lhx3 complex from bioinformatics analysis of these ChIP-seq datasets, we identified only *Arhgap36*, rather than a cluster of HH-signaling components, whose function has been implicated in Shh signaling pathway (Rack et al., 2014). We identified the binding peak in the promoter region of *Arhgap36* (Figure 4A). Within the binding site, we discovered a motif similar to the previously defined consensus HxRE (for hexamer response element) (Figure 4A and B), which is the binding site for the Isl1-Lhx3 complex (Lee et al., 2013; Lee et al., 2008). To test whether the Isl1-Lhx3 complex is recruited to the HxRE of the *Arhgap36* gene in vivo, we performed ChIP assay with antibodies against Isl1 and Lhx3 using E12.5 mouse embryonic spinal cord extracts. Both Isl1 and Lhx3 strongly bound to the genomic region of the *Arhgap36* gene containing the ChIP-seq peak while they showed much weaker binding to a negative control genomic region *Untr6* (Mali et al., 2008) (Figure 4C). These results indicate that the endogenous Isl1-Lhx3 complex is recruited to the *Arhgap36* gene in the developing spinal cord.

### *Arhgap36* HxRE is activated by the Isl1-Lhx3 complex

To determine whether ARHGAP36 expression is induced directly by Isl1-Lhx3 complex via the HxRE within the ChIP-seq peak, we constructed a luciferase reporter and a GFP reporter linked to two copies of the genomic fragment containing the HxRE in the peak (herein named *Arhgap36*-enh) (Figure 4B). In mouse embryonic P19 cells, co-expression of Isl1 and Lhx3, which form the Isl1-Lhx3 complex with endogenous NLI, strongly activated the luciferase reporter, whereas Isl1 or Lhx3 alone showed only marginal to no activations (Figure 4D). To test whether Isl1-Lhx3 complex can activate the *Arhgap36*-enh in vivo, we electroporated the chick neural tube with a GFP reporter linked to two copies of the *Arhgap36*-enh at a time when MNs are being specified, and found that GFP is specifically expressed in MNs (Figure 4E, upper panels). When we co-electroporated Isl1 and Lhx3 expression vectors with the GFP reporter, *Arhgap36*-enh was ectopically activated in the dorsal spinal cord (Figure 4E, lower panels), coincident with the occurrence of Hb9^+^ ectopic MNs (Figure 4E and F). As a negative control experiment, TATA-GFP construct containing no HxRE was electroporated into the chick neural tube and this GFP reporter was not activated even when ectopic Hb9 is induced by the expression of Isl1 and Lhx3 in the dorsal spinal cord (Figure 4E). Together, these results indicate that the Isl1-Lhx3 complex directly triggers the expression of ARHGAP36 via the HxRE motif in the *Arhgap36* gene during MN differentiation.

### ARHGAP36 is expressed in developing spinal MNs

**Figure 5.**
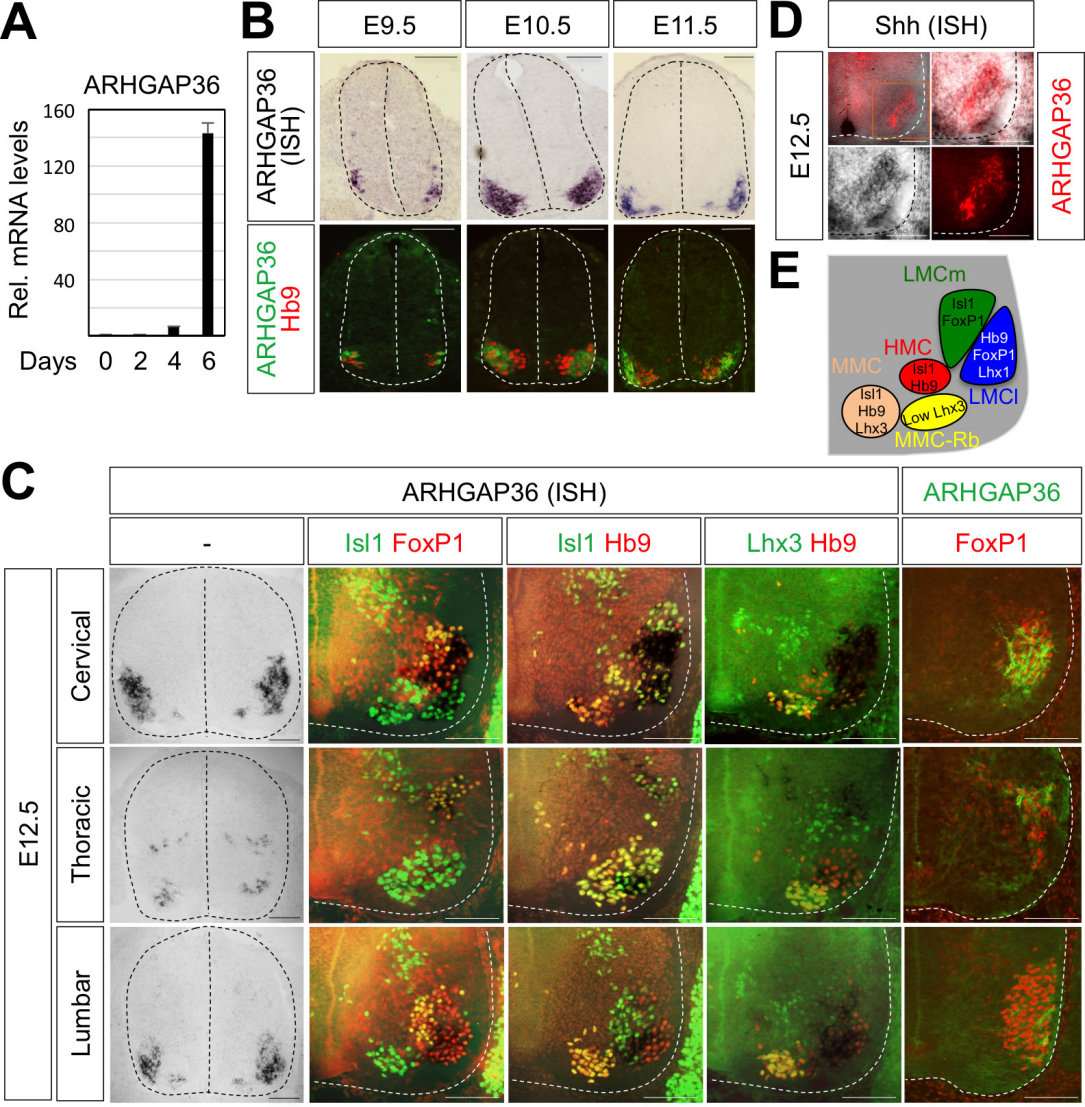
Expression of ARHGAP36 in the developing spinal MNs. (**A**) Induction of ARHGAP36 expression in MNs differentiated from mouse ESCs was determined by qRT-PCR. Relative expression levels are shown as the mean of duplicate values obtained from representative experiments. Error bars represent standard deviation. (**B,C**) ARHGAP36 was specifically expressed in MNs of mouse embryos at E9.5, E10.5, E11.5 and E12.5 stages, as shown by ISH with a probe detecting ARHGAP36 and IHC for ARHGAP36, Isl1/FoxP1, Isl1/Hb9, Lhx3/Hb9 and FoxP1. From E12.5 and onward, ARHGAP36 expression was highly enriched in LMCl (Isl1^-^/FoxP1^+^) region, some in MMC-rhomboideus (Rb) (Hb9^+^/Lhx3^low^) and a very little in the most medial part of MMC but not in LMCm (Isl1^+^/FoxP1^+^) at cervical level. ARHGAP36 is also expressed in PGC (FoxP1^+^/Isl1^+^) and HMC (Isl1^+^/Hb9^+^) neurons at thoracic level but with relatively lower expression compared to the cervical level. At lumbar level, ARHGAP36 is enriched in LMCl (Isl1^-^/FoxP1^+^) of the spinal cord. Scale bars: 100 μm. (**D**) Co-localization of ARHGAP36 with Shh shown by ISH of Shh and IHC of ARHGAP36 in mouse E12.5 spinal cord at cervical level. Shh is co-localized with ARHGAP36 mostly in LMCl region in mouse spinal cord. Scale bars: 100 μm. (**E**) Schematic drawing shows the LMCm, LMCl, HMC, MMC and MMC-rhomboideus (Rb) motor columns in the ventral spinal cord with representative markers. 10.7554/eLife.46683.014Figure 5—source data 1.Source data for Figure 5A.

The binding of Isl1-Lhx3 complex to the *Arhgap36* gene raises the possibility that the expression of ARHGAP36 is induced as MNs become specified in the developing spinal cord. In support of this idea, the expression of ARHGAP36 was induced when MNs were derived from mouse embryonic stem cells (mESCs) under MN differentiation condition (Lee et al., 2012; Wichterle et al., 2002) (Figure 5A). To further test this possibility, we performed ISH and IHC on mouse embryonic spinal cord. Consistent with the finding that Isl1-Lhx3 triggers the expression of ARHGAP36, ARHGAP36 began to be expressed in newborn MNs around E9.5 (Figure 5B) and its expression was strongly induced in MNs at E10.5-E11.5 (Figure 5B) along the rostrocaudal axis of the spinal cord, which is soon after Isl1^+^/Lhx3^+^ MNs are born. From E12.5, the expression of ARHGAP36 is most highly enriched in LMCl (Isl1^-^/FoxP1^+^) region, some in MMC-rhomboideus (Hb9^+^/Lhx3^low^) and a very little in the most medial part of MMC but not in LMCm (Isl1^+^/FoxP1^+^) at cervical level. At thoracic level, ARHGAP36 is also expressed in preganglionic motor column (PGC) (FoxP1^+^/Isl1^+^) and HMC (Isl1^+^/Hb9^+^) neurons but with relatively lower expression compared to the cervical level. At lumbar level, ARHGAP36 is relatively highly enriched in LMCl (Isl1^-^/FoxP1^+^) and show very low level in the most medial part of MMC (Figure 5C and E). To examine the co-localization of ARHGAP36 with Shh, we performed ISH of Shh and IHC of ARHGAP36 in mouse E12.5 spinal cord at cervical level. Shh is co-localized with ARHGAP36 mostly in LMCl region in mouse spinal cord (Figure 5D). FoxP1 is expressed high in LMC region at brachial and lumbar levels as well as in PGC region at thoracic level, which is co-expressed in all ARHGAP36^+^ cells. Also ARHGAP36 protein was mainly localized in the cytoplasm (Figure 5B and C), suggesting that ARHGAP36 protein might function as a modulator of a cytoplasmic signaling cascade within MNs. We also examined the expression of Arhgap36 in chick embryo and found that it is ubiquitously expressed within the spinal cord but not in other tissues (Figure 7—figure supplement 3A).

### Shh pathway is activated by ARHGAP36 expression in spinal cord

**Figure 6.**
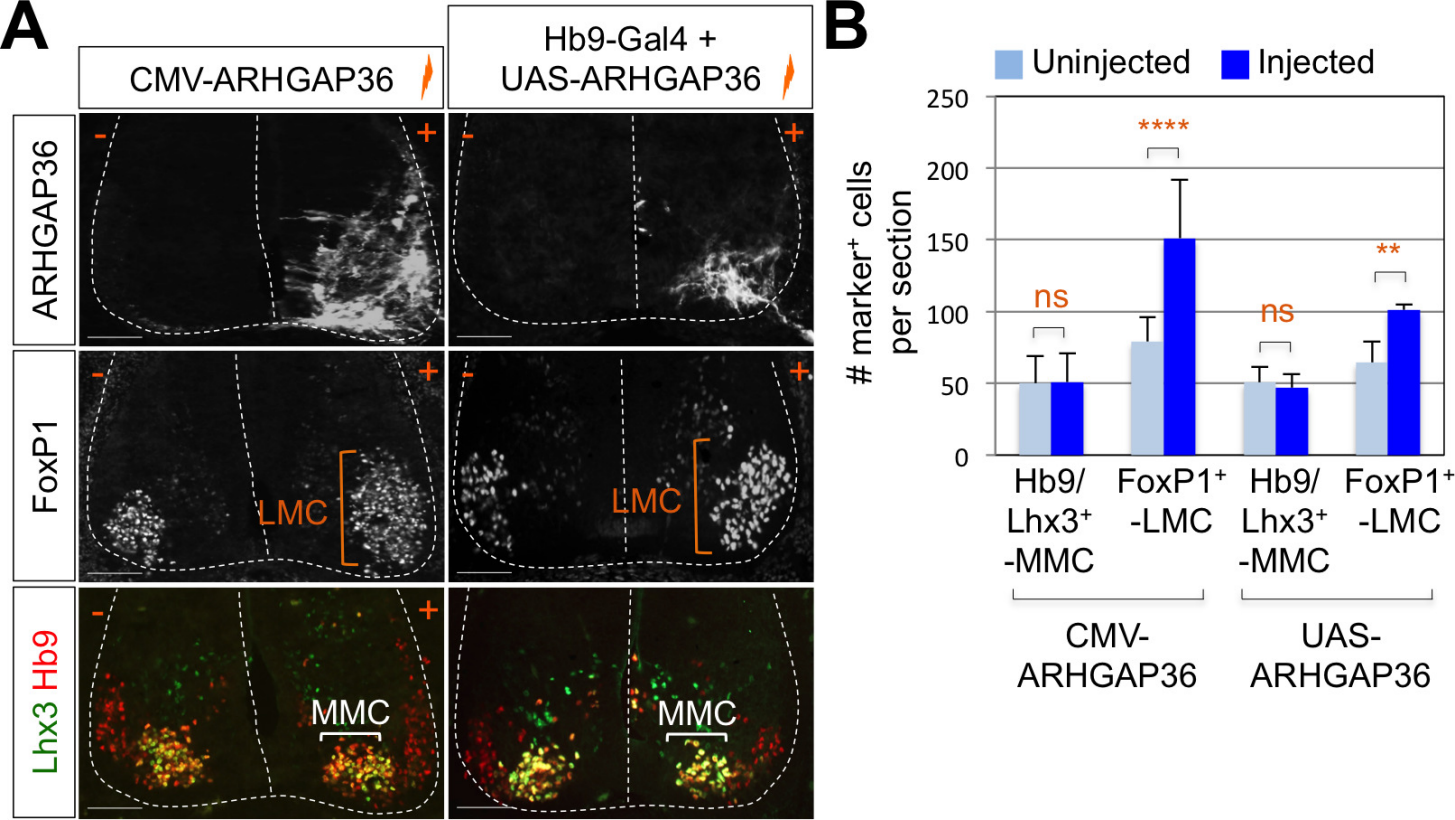
Expression of ARHGAP36 promotes LMC specification in developing chick spinal cord. (**A**) ARHGAP36 constructs were injected and electroporated in chick neural tube and embryos (n = 8 ~ 15) were harvested 4 days post electroporation (four dpe). Ectopic expression of ARHGAP36 driven by CMV promoter in most injected cells induced robust expression of FoxP1^+^ LMC neurons (orange bracket) in ventral spinal cord but had no effect on MMC (Hb9^+^/Lhx3^+^) neurons (white bracket). Targeting the expression of ARHGAP36 specifically in motor neurons using Hb9-Gal4/UAS-ARHGAP36 system also lead to the robust induction of FoxP1^+^ LMC neurons (orange bracket) but had no effect on MMC (Hb9^+^/Lhx3^+^) neurons (white bracket). +, electroporated side; -, non-electroporated control side. Experiments were repeated independently at least three times. Scale bars: 100 μm. (**B**) Quantification of the number of FoxP1^+^ neurons and MMC (Hb9^+^/Lhx3^+^) neurons on the electroporated (+) and non-electroporated (-) sides of the spinal cord. Data are mean ± s.d. **p<0.001, ****p<0.00001; ns, non-significant (Student’s t-test). n = 6 ~ 20 independent images per each sample. 10.7554/eLife.46683.019Figure 6—source data 1.Source data for Figure 6B.

To test whether ARHGAP36 is able to mediate Shh activity within the developing spinal cord, we have ectopically expressed ARHGAP36 in the neural tube using *in ovo* electroporation and examined the expression pattern of MN genes as well as genes in spinal progenitor domain and Shh pathway by IHC and ISH. ARHGAP36 misexpression resulted in a strong ventralization of the dorsal spinal cord (Figure 6—figure supplement 1A), mimicking Shh activity. Apparently, the size of the spinal cord was increased with ARHGAP36 expression and MN genes such as *Hb9, Isl1/2* and *Slc18a3* were highly upregulated in the electroporated side by ARHGAP36 (Figure 6—figure supplement 1A). Nkx2.2, a marker for p3 domain (V3 interneuron progenitors), and Olig2, a marker for pMN, were also ectopically expressed in the electroporated side (Figure 6—figure supplement 1A). As the effect of ARHGAP36 expression highly resembled that of activated Shh pathway, we examined two downstream target genes of Shh pathway, *Ptch1* and *Gli1*. Both genes were also upregulated in the dorsal spinal cord, demonstrating activation of Gli-dependent transcription (Figure 6—figure supplement 1A). Given the requirement of Shh in LMC and the specific expression of ARHGAP36 in LMC neurons among mature MNs, we considered the possibility that ARHGAP36 has a role in LMC formation. When ARHGAP36 is misexpressed preferentially in ventral spinal cord and harvested at 4 days post electroporation (dpe), the number of FoxP1^+^ LMC neurons increased drastically, but there was no change in MMC (Hb9^+^/Lhx3^+^) neurons (Figure 6A and B). To avoid the defects in proliferation and neural patterning possibly caused by the aberrant activation of Shh in the neural progenitors, we adopted Gal4/UAS system to drive the motor neuron specific expression of ARHGAP36. Under this condition, ARHGAP36 was expressed specifically in postmitotic MNs and resulted in significant increase of FoxP1^+^ LMC neurons but had no effect on MMC (Hb9^+^/Lhx3^+^) neurons (Figure 6A and B). These data suggest that ARHGAP36 is sufficient to direct LMC fate-determination.

**Figure 6—figure supplement 1.**
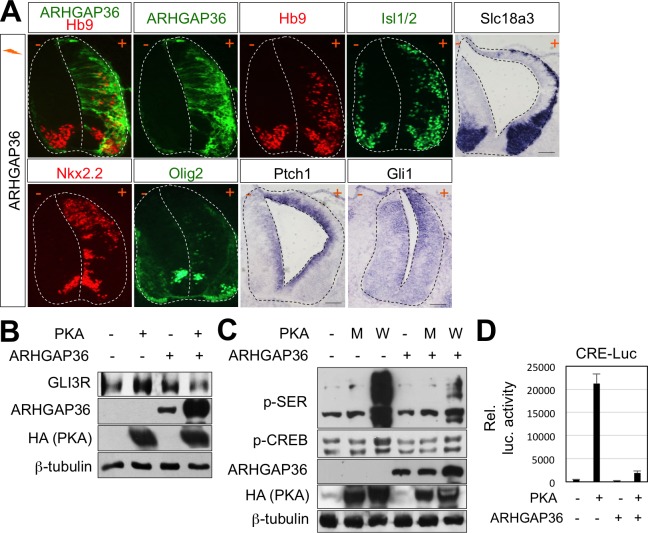
Activation of Shh pathway by ARHGAP36 expression in spinal cord. (**A**) *In ovo* electroporation of ARHGAP36 construct. ARHGAP36 construct was injected and electroporated in chick neural tube and embryos (n = 8 ~ 12) were harvested 3 days post electroporation (three dpe). Ectopic expression of ARHGAP36 induced expression of ventral spinal neuronal genes including motor neuron genes (Hb9, Isl1/2, Slc18a3), ventral progenitor genes (Nkx2.2 and Olig2) and downstream target genes of Shh pathway (Ptch1 and Gli1) as shown by ISH and IHC. +, electroporated side; -, non-electroporated control side. Experiments were repeated independently at least three times. Scale bars: 100 μm. (**B**) Western blot analysis showed that ARHGAP36 inhibits PKA activity. PKA expression resulted in producing Gli3R, repressor form of Gli3 (lane 2) in HEK293T cells. Co-expression of ARHGAP36 blocks the formation of Gli3R by PKA (lane 4). β-tubulin was used as a loading control. (**C**) ARHGAP36 inhibited the level of phosphorylation of putative PKA substrates shown by western blot with anti-p-SER antibody and the level of phospho-CREB (p-CREB), a known PKA target in NIH3T3 cells. W indicates PKA wild type and M indicates PKA kinase dead mutant (K73H). β-tubulin was used as a loading control. (**D**) Luciferase reporter assay in HEK293T cells. The activation of CRE-luc is directed by PKA-phosphorylated CREB and this activity was blunted by co-expression of ARHGAP36, indicating that ARHGAP36 inhibits the kinase activity of PKA. Data are mean ± s.d. 10.7554/eLife.46683.017Figure 6—figure supplement 1—source data 1.Source data for Figure 6—figure supplement 1D.

**Figure 6—figure supplement 2.**
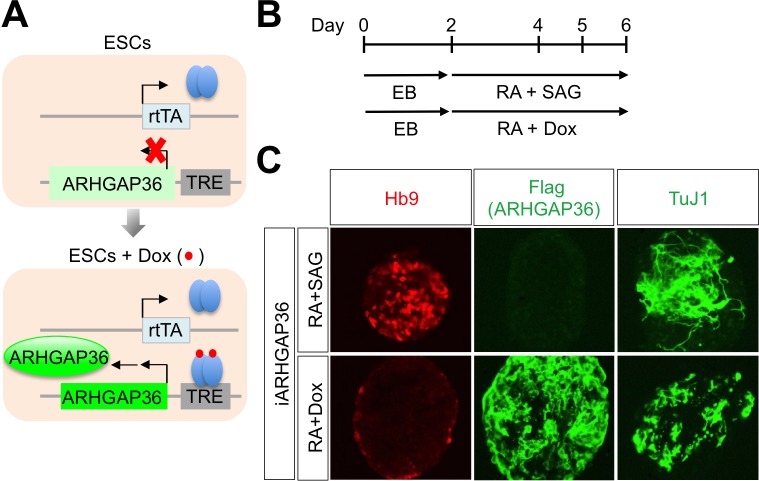
ARHGAP36 is not sufficient to induce MN differentiation in mESCs. (**A**) Schematic representation of inducible ARHGAP36-ESCs before and after treatment with Dox (Doxycycline). TRE, tetracycline response element; rtTA, reverse tetracycline transactivator. (**B**) Illustration of experimental design to differentiate ESCs to MNs. RA and SAG or RA and Dox were treated at day two and EBs were harvested at day six for further analyses. EB, embryoid body; RA, retinoic acid; SAG, Smoothened agonist. (**C**) IHC analyses in ESC-derived MNs cultured with RA +SAG or RA +Dox at differentiation day 6. Hb9 labels MNs and it was induced only by RA +SAG. Treatment of RA +Dox induced ARHGAP36 and pan-neuronal marker TuJ1, but not MN marker Hb9.

### PKA activity is inhibited by ARHGAP36

To further test whether ARHGAP36 functions through Shh, we examined the effects of ARHGAP36 on Gli3 processing in cells, which is modulated by PKA in response to Shh (Pan et al., 2009). In support of the idea that the expression of ARHGAP36 in chick spinal cord induces Gli-dependent transcription through blocking PKA activity, ARHGAP36 blunted the activity of PKA from producing Gli3R, the repressor form of Gli3 (Figure 6—figure supplement 1B, lane 4). We then examined whether the kinase activity of PKA was inhibited by ARHGAP36 by measuring the level of phosphorylated CREB (Nichols et al., 1992), a direct target of PKA as well as phospho serine (pSER) levels of putative PKA substrates in the whole cell lysate. WT PKA, but not a kinase dead mutant form of PKA (K73H) (Iyer et al., 2005; Zhong et al., 1997), showed a robust increase in pSER and p-CREB levels (Figure 6—figure supplement 1C, lanes 2, 3). The pSER and p-CREB levels in the presence of WT PKA were drastically reduced by ARHGAP36 overexpression (Figure 6—figure supplement 1C, lane 6). PKA phosphorylates CREB, and p-CREB in turn binds and activates CRE-luciferase reporter (Nichols et al., 1992; Grewal et al., 2000). Indeed, the CRE-Luc reporter was strongly activated by PKA, but this activation was blunted by co-expression of ARHGAP36 in HEK293T cells (Figure 6—figure supplement 1D). Taken together, our data indicate that ARHGAP36 inhibits PKA and de-represses Gli activity.

### ARHGAP36 alone is not sufficient to induce MNs from mouse embryonic stem cells

As ARHGAP36 has a potent activity in Shh signaling stimulation and MN induction in chick spinal cord, we tested whether ARHGAP36 alone is sufficient in inducing MNs from mouse embryonic stem cells (mESCs). We generated a mouse ESC line, in which doxycycline (Dox) induces the expression of ARHGAP36 (iARHGAP36-ESCs) and tested whether ARHGAP36 can replace the activity of Shh (Figure 6—figure supplement 2). The iARHGAP36-ESCs enabled us to control the exact timing of ARHGAP36 expression by treating the cells with Dox (Figure 6—figure supplement 2A). We used conventional MN differentiation method with retinoic acid (RA) and Shh agonist (Smoothened agonist, SAG) to compare the efficiency of MN generation (Figure 6—figure supplement 2B). iARHGAP36-ESCs treated with RA and SAG exhibited effective MN differentiation, as determined by the induction of MN markers such as Hb9. iARHGAP36-ESCs treated with RA and Dox without SAG differentiated into neurons as marked by TuJ1 expression, but failed to induce the MN gene, Hb9 (Figure 6—figure supplement 2C), suggesting that ARHGAP36 alone is not sufficient to activate Shh downstream pathway to promote the initial ventralization and MN induction in mESCs. These results suggest that Shh ligand is likely needed for ARHGAP36 to function properly in vivo.

### ARHGAP36 mediates the positive effect of AKT in Shh signaling

**Figure 7.**
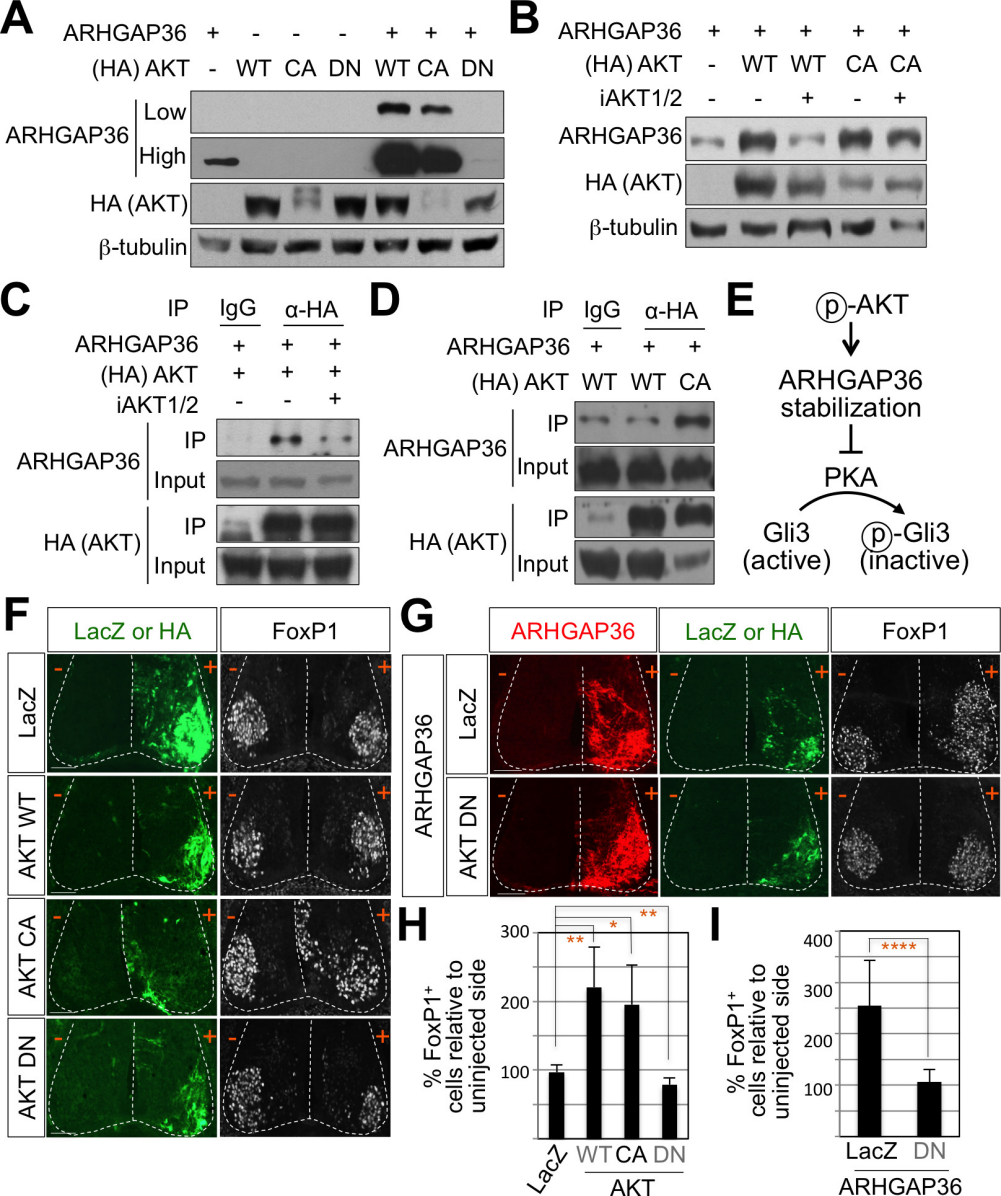
AKT potentiates Shh signaling through stabilization of ARHGAP36 proteins and AKT-ARHGAP36 axis is required for LMC specification. (**A**) ARHGAP36 was stabilized dramatically by AKT WT and CA, but not by DN in HEK293T cells. ARHGAP36 was transiently transfected with AKT constructs in HEK293T cells and the protein levels were monitored by western blotting. β-tubulin was used as a loading control. (**B**) 10 μM of AKT inhibitor (iAKT1/2) was treated for 20 hr and the protein level of ARHGAP36 was monitored. AKT inhibitor reversed the effect of AKT WT in stabilizing ARHGAP36 protein but had no effect on constitutively active form of AKT. (**C**) Co-immunoprecipitation assay with HEK293T cells transiently transfected with the expression vectors for HA-tagged AKT and ARHGAP36 showed that AKT WT co-purified ARHGAP36, and this interaction was decreased by iAKT1/2, the AKT inhibitor. (**D**) The CA form of AKT interacted with ARHGAP36 more robustly than AKT WT. ARHGAP36 with either HA-tagged AKT WT or AKT CA was transfected into HEK293T cells and immunoprecipitated with anti-HA antibody that pull-downs AKT. Anti-IgG antibody was used as a negative control. (**E**) Illustration of the modulatory pathway showing that activated AKT stabilizes ARHGAP36 proteins, which in turn blocks the kinase activity of PKA, which results in Gli-dependent transcriptional activation via dephosphorylation of Gli. (**F**) IHC analyses in the chick neural tube electroporated with AKT WT, CA and DN. Embryos (n = 8–10) were harvested 4dpe. AKT WT or CA increased the number of FoxP1^+^ cells by almost two fold in the electroporated side (+) compared to the non-electroporated control side (-). Experiments were repeated independently at least three times. Scale bars: 100 μm. (**G**) The analysis of ectopic FoxP1^+^ neuron formation by ARHGAP36 in the presence of either AKT DN or LacZ in the chick neural tube. Embryos (n = 8–10) were harvested 4dpe. +, electroporated side; -, non-electroporated control side. AKT DN completely blocked the effect of ARHGAP36 in inducing ectopic FoxP1 expression in the electroporated cells. Experiments were repeated independently at least three times. Scale bars: 100 μm. (**H,I**) Quantification of the number of FoxP1^+^ neurons on the electroporated (+) and non-electroporated (-) sides of the spinal cord. Data are mean ± s.d. *p<0.01, **p<0.001, ****p<0.00001 (Student’s t-test). n = 6 ~ 27 independent images per each sample. 10.7554/eLife.46683.025Figure 7—source data 1.Source data for Figure 7H and 7I.

To fully understand the nature of ARHGAP36 function, we tried to identify signaling pathways that regulate the activity of ARHGAP36 through post-translational modifications, including phosphorylation. We adopted GPS 3.0 website for predicted sites based on protein sequences (Xue et al., 2005). We found several predicted phosphorylation sites in ARHGAP36 proteins, and AKT kinase was one of the high ranked kinase (data not shown). Phosphoinositide 3-kinase (PI3-kinase)-dependent AKT activation plays an essential role in Shh signaling by antagonizing PKA-mediated Gli inactivation in the specification of neuronal fates in chicken neural explants (Riobó et al., 2006). To test whether ARHGAP36 functions as a downstream effector of AKT, we transfected ARHGAP36 with AKT constructs in HEK293T cells and measured the protein levels of ARHGAP36 in the presence of AKT. As ARHGAP36 is not expressed endogenously in HEK293T cells, ARHGAP36 proteins are expressed only from the ARHGAP36-encoding plasmid, in which CMV promoter drives the transcription of ARHGAP36. Interestingly, wild type AKT (WT) and constitutively active myristoylated form of AKT (CA), but not a nonphosphorylatable dominant negative form of mutant AKT (DN), stabilized ARHGAP36 proteins robustly (Figure 7A), suggesting that AKT increases ARHGAP36 proteins likely by stabilizing ARHGAP36 protein, rather than activating the ARHGAP36 promoter transcriptionally. We also found that the half-life of ARHGAP36 protein, treated with cycloheximide that blocks the protein translation, was prolonged in the presence of AKT (Figure 7—figure supplement 1). This stabilization of ARHGAP36 protein by AKT WT was reversed by AKT inhibitor, but the CA form of AKT was not affected by AKT inhibitor (Figure 7B). Also AKT and ARHGAP36 associated with each other in co-immunoprecipitation assays and this association was decreased by treatment of AKT inhibitor (Figure 7C). The CA form of AKT interacted with ARHGAP36 more robustly than WT AKT (Figure 7D). These results show that activated AKT interacts with ARHGAP36 and stabilizes ARHGAP36 proteins (Figure 7E). It needs to be further confirmed whether ARHGAP36 interacts with AKT directly and is a genuine substrate of AKT.

**Figure 7—figure supplement 1.**
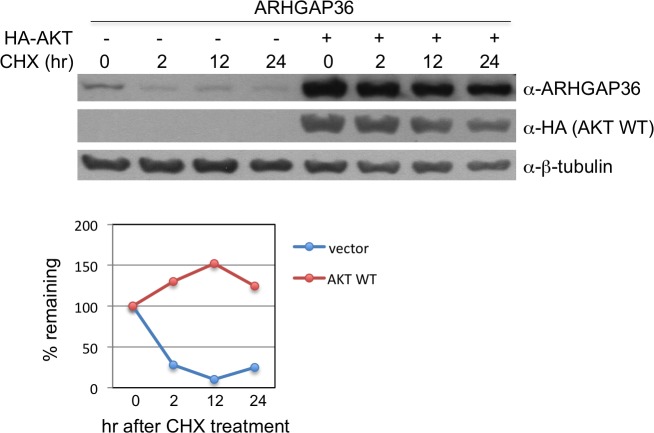
AKT stabilizes protein level of ARHGAP36. Measurement of half-life of ARHGAP36 in HEK293T cells transfected with empty vector or vector encoding AKT WT, following treatment with 200 ng/ml of the protein synthesis inhibitor cycloheximide (CHX). The half-life of ARHGAP36 was roughly estimated to ~1 hr for vector alone, while it was not degraded within 24 hr for AKT WT. These experiments were repeated multiple times, and we obtained similar results. 10.7554/eLife.46683.022Figure 7—figure supplement 1—source data 1.Source data for Figure 7—figure supplement 1.

**Figure 7—figure supplement 2.**
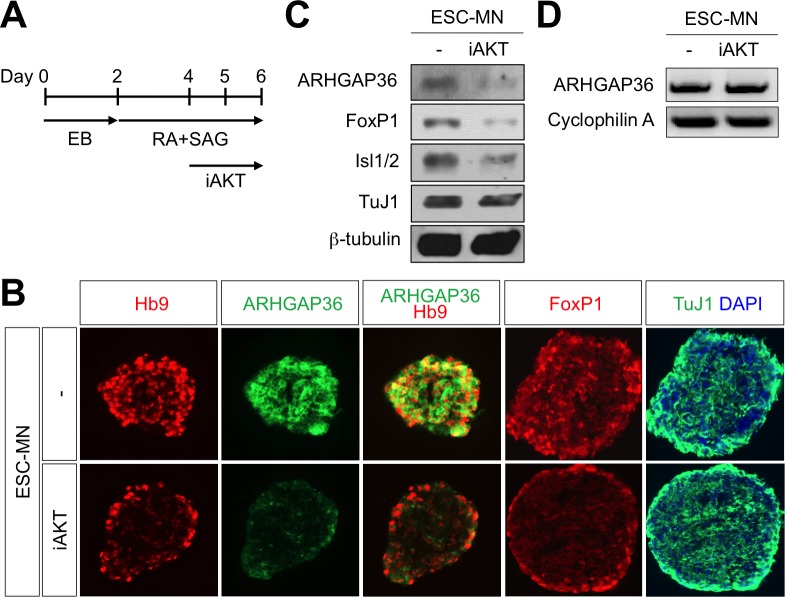
AKT inhibition blocks MN differentiation from mouse ESCs. (**A**) Illustration of experimental design to differentiate ESCs to MNs. RA and SAG were treated at day two and AKT inhibitor was treated from day four and EBs were harvested at day six for further analyses. EB, embryoid body; RA, retinoic acid; SAG, Smoothened agonist. (**B**) IHC analyses in ESC-derived MNs cultured with RA +SAG with or without iAKT at differentiation day 6. Hb9 labels MNs. ARHGAP36 was induced in MN differentiation condition. Treatment of iAKT reduced expression of ARHGAP36, FoxP1 and Hb9 but not pan-neuronal marker TuJ. (**C**) Western blotting analysis showed reduced expression of ARHGAP36, FoxP1 and Isl1/2 but not pan-neuronal marker TuJ by AKT inhibitor treatment. (**D**) RT-PCR analysis revealed that ARHGAP36 mRNA level was not affected by AKT inhibition. Cyclophilin A was used as a loading control.

**Figure 7—figure supplement 3.**
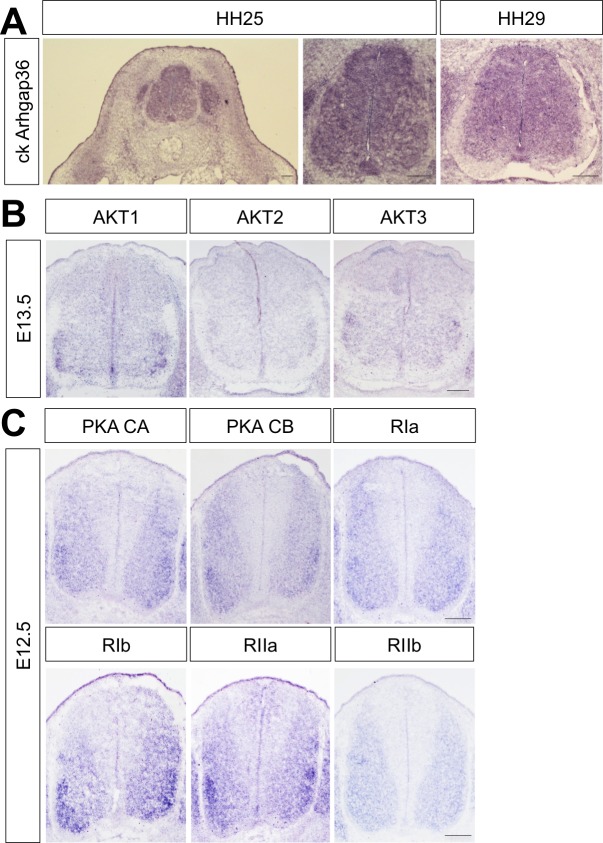
Expression of Arhgap36 in chick spinal cord and AKTs and PKAs in developing mouse spinal cord. (**A**) ISH for Arghap36 in HH25 and HH29 chick embryos showing its broad expression in the spinal cord at brachial level. (**B**) ISH for AKT1, AKT2 and AKT3 in E13.5 mouse embryos showing their expression in ventro-lateral region of the spinal cord at cervical level. (**C**) ISH for the PKA catalytic subunits CA and CB and the regulatory subunits RIa, RIb, RIIa and RIIb in E12.5 mouse embryos showing their expression in ventro-lateral region of the spinal cord at cervical level. Scale bars: 100 μm.

### AKT is required for MN differentiation in mouse ESCs

AKT is a key player in activation of MN survival pathways after spinal cord injury (Yu et al., 2005) and it is downregulated in amyotrophic lateral sclerosis (ALS) (Peviani et al., 2014), suggesting that AKT may also play a role in embryonic MN development. Given that ARHGAP36 was induced when mESCs differentiated into MNs (Figure 5A), we hypothesized that AKT regulates the protein levels of ARHGAP36 affecting the efficiency of MN differentiation from mESCs. We used the MN differentiation condition with RA and SAG, a Smoothened agonist that stimulates Shh pathway, followed by treatment with AKT inhibitor for 2 days (Figure 7—figure supplement 2A) and harvested differentiated EBs for immunostaining (Figure 7—figure supplement 2B) and immunoblotting (Figure 7—figure supplement 2C) to monitor MN differentiation. Treatment of AKT inhibitor decreased ARHGAP36 protein levels as well as MN markers such as Isl1/2, FoxP1 and Hb9 but not pan-neuronal marker TuJ1 (Figure 7—figure supplement 2B and C). AKT inhibitor did not affect the mRNA level of ARHGAP36 (Figure 7—figure supplement 2D). These results suggest that AKT activity plays an important role in MN differentiation likely through modulating the level of ARHGAP36 proteins.

### AKT-ARHGAP36 axis modulates Shh signaling in LMC specification

To further investigate the roles of AKT in modulating Shh signaling in LMC specification, we examined the expression pattern of AKTs using ISH. AKT1, AKT2, and AKT3 showed relatively low expression in the spinal cord but they were specifically enriched in the lateral region of the spinal cord (Figure 7—figure supplement 3B). We also examined the expression patterns of PKA catalytic isoforms and regulatory isoforms using ISH. Most of them were expressed in the lateral region of the spinal cord, while PKA CA, CB, RIb and RIIa were more enriched in the LMC region (Figure 7—figure supplement 3C). Given the relatively high expression of AKT and PKA in ventro-lateral region of the spinal cord and the role of Shh in inducing the activation of AKT in cell lines such as LIGHT cells and HUVEC cells (Kanda et al., 2003; Riobó et al., 2006), we proposed that Shh expressed in the motor neurons triggers AKT activation, which in turn stabilizes the protein level of ARHGAP36 in LMC neurons. Indeed, we detected reduced expression of ARHGAP36 in *Shh*-cKO (Figure 3—figure supplement 1A) suggesting that the protein level of ARHGAP36 can be modulated through AKT activation by Shh in LMC neurons of developing mouse spinal cord. To test the activity of AKT in inducing FoxP1^+^ LMC MNs, we injected WT, CA and DN form of AKT in chick spinal neural tube and monitored the expression of FoxP1. Interestingly, AKT WT and CA increased the number of cells expressing FoxP1 by almost two fold in the electroporated side of the spinal cord compared to the non-electroporated side (Figure 7F and H), while AKT DN resulted in further reduction of endogenous FoxP1 in LMC region (Figure 7F and H). Furthermore, this AKT DN actively blocked the effect of ARHGAP36 in inducing ectopic FoxP1 in the electroporated cells (Figure 7G and I), suggesting that AKT is required for the ARHGAP36 to function as a modulator of Shh signaling in LMC specification. Taken together, our results demonstrate that AKT-directed maintenance of physiological levels of ARHGAP36 is likely critical for effectively activating Shh signaling through inhibition of PKA in LMC MN formation.

### Requirement of ARHGAP36 for LMC formation in mice

**Figure 8.**
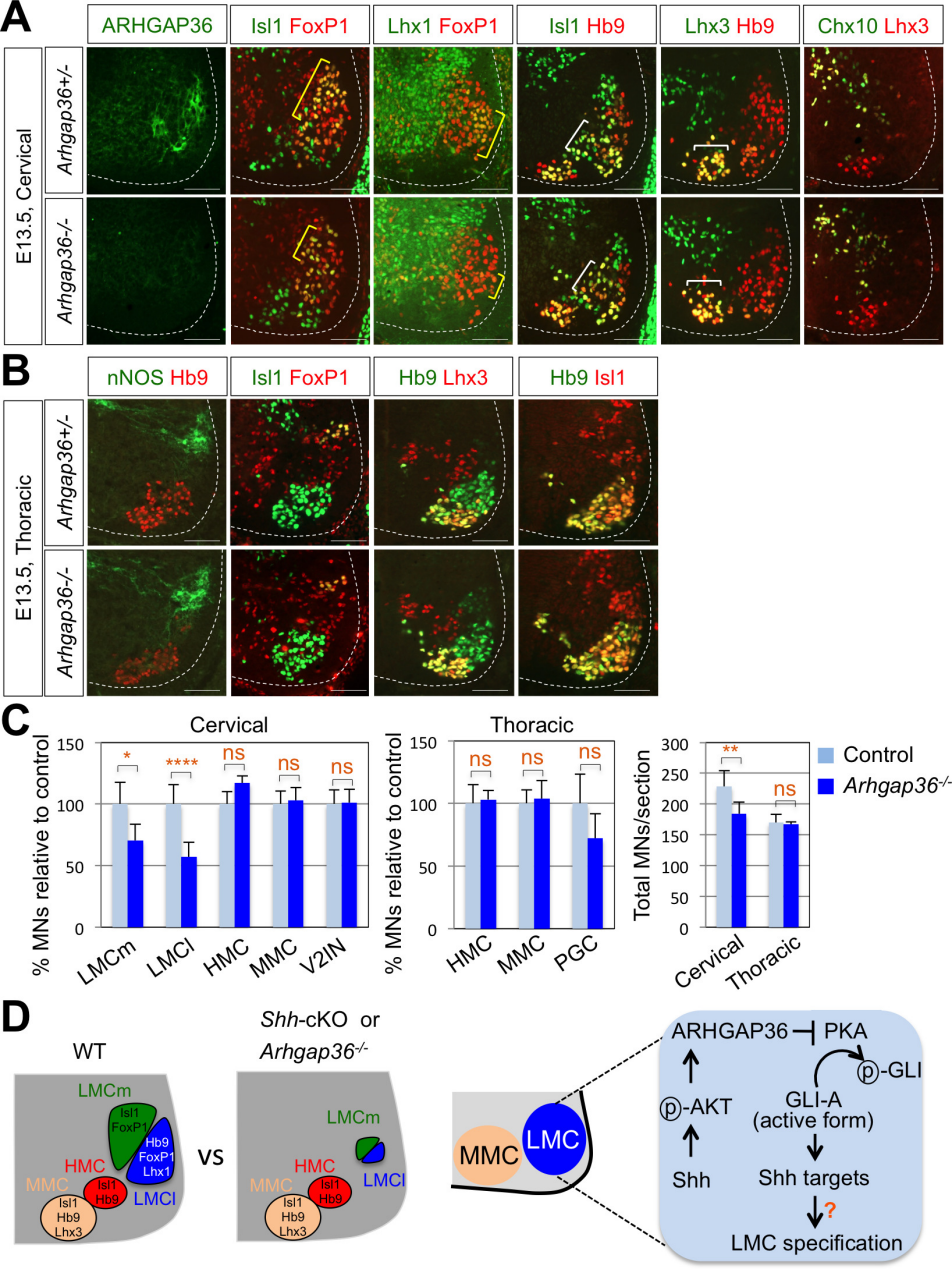
ARHGAP36 is required for LMC formation in mice. (**A**) IHC analyses of E13.5 *Arhgap36*^-/-^ mutant embryo (n = 4) (lower panel) and their littermate controls (n = 5) (upper panel). Ventrolateral quadrants of the cervical level of spinal cord are shown in all panels. IHC with anti-ARHGAP36 antibody confirms the absence of ARHGAP36 expression in *Arhgap36-*null embryos. LMCm (Isl1^+^/FoxP1^+^) and LMCl (Lhx1^+^/FoxP1^+^) neurons were significantly reduced in *Arhgap36*^-/-^. On the other hand, the numbers of MMC (Hb9^+^/Lhx3^+^), HMC (Isl1^+^/Hb9^+^) neurons and V2-INs (Lhx3^+^/Chx10^+^) did not change. Scale bars: 100 μm. (**B**) At thoracic level, there was no difference in PGC (nNOS^+^ or Isl1^+^/FoxP1^+^), HMC (Hb9^+^/Isl1^+^), and MMC (Hb9^+^/Lhx3^+^) neurons compared to control littermates. Scale bars: 100 μm. (**C**) Quantification of the number of LMCm (Isl1^+^/FoxP1^+^), LMCl (Lhx1^+^/FoxP1^+^), MMC (Hb9^+^/Lhx3^+^), HMC (Isl1^+^/Hb9^+^) and V2-INs (Lhx3^+^/Chx10^+^) at cervical level in E13.5 mouse embryonic spinal cord. Data are mean ± s.d. *p<0.01, **p<0.001, ****p<0.00001; ns, non-significant; n = 6 ~ 12 independent images per each sample. (**D**) Proposed model. In *Shh*-cKO or *Arhgap36^-/-^* mutant embryos, LMCm and LMCl neurons are reduced with no expansion of other motor columns, and thus this results in the reduction of total MNs compared to WT control. AKT, activated in response to Shh, stabilizes ARHGAP36 protein, which in turn inhibits the kinase activity of PKA. This results in Gli-dependent transcriptional activation and LMC formation in MNs at cervical level of the spinal cord. 10.7554/eLife.46683.031Figure 8—source data 1.Source data for Figure 8C.

To further define the roles of ARHGAP36 in developing mouse embryos, we generated *Arhgap36* deficient mice using Clustered Regularly Interspaced Short Palindromic Repeats (CRISPR) and CRISPR-associated proteins (Cas) system (Figure 8—figure supplement 1A and B) (Yang et al., 2014; Ran et al., 2013; Jinek et al., 2012). By IHC with anti-ARHGAP36 antibody, we confirmed that ARHGAP36 proteins were not expressed in *Arhgap36^-/-^* and *Arhgap36^-/y^* mutant spinal cord at different time points (Figure 8A and Figure 8—figure supplement 2A). We then examined the specification of MNs, especially formation of LMC MNs at cervical level where ARHGAP36 is highly expressed (Figure 5B and C). At earlier stages of mouse embryo from E9.5 to 11.5, there was no obvious defect in proliferation of neural progenitor cells, ventral neural patterning and overall MN generation (Figure 8—figure supplement 1C). At E12.5, the overall number of MNs in *Arhgap36^-/-^* mutant spinal cord was similar to that of control littermates and the total number of FoxP1^+^ MNs at cervical level was not affected either (Figure 8—figure supplement 1D). However, we found that LMCm (Isl1^+^/FoxP1^+^) neurons are increased, whereas LMCl (Lhx1^+^/FoxP1^+^) neurons are decreased in *Arhgap36^-/-^* mutant spinal cord (Figure 8—figure supplement 1D). Concomitantly, there was an increase in cleaved Caspase3 positive apoptotic cells in the absence of ARHGAP36 (Figure 8—figure supplement 1D). LMCl neurons are born later than LMCm neurons, and these LMCl neurons have to migrate through earlier born LMCs. These results suggest that later born presumptive LMCl neurons may fail to fully differentiate into LMCl neurons and, instead, either adopt LMCm characteristics or undergo cell death in the absence of ARHGAP36. Intriguingly, from E13.0, the numbers of LMCm (Isl1^+^/FoxP1^+^) and LMCl (Lhx1^+^/FoxP1^+^) neurons were reduced in *Arhgap36^-/-^* spinal cord (Figure 8A and C), which may be caused by the increased cell death (Figure 8—figure supplement 1D). These results suggest that ARHGAP36 is required for proper generation or maintenance of LMC MNs. In contrast, there was no significant difference in MMC (Hb9^+^/Lhx3^+^), HMC (Hb9^+^/Isl1^+^) MNs and V2-INs (Chx10^+^/Lhx3^+^) (Figure 8A and C). At thoracic levels, nNOS^+^ PGC, HMC (Hb9^+^/Isl1^+^) and MMC (Hb9^+^/Lhx3^+^) MNs in *Arhgap36* deficient spinal cord were expressed comparably to those in control littermate (Figure 8B). *Arhgap36* gene is located in the X chromosome and we examined whether there is phenotypic difference between male and female mutant embryos. While there was no significant difference in MMC (Hb9^+^/Lhx3^+^) and nNOS^+^ PGC MNs between sexes, the number of FoxP1^+^ LMC neurons at cervical level was reduced in female mutant embryo (Figure 8) but not in male mutant embryo at E13.5 (Figure 8—figure supplement 2). The molecular basis underlying this sexual dimorphism needs to be further investigated in the future. Taken together, these data support the notion that specific expression of ARHGAP36 in a subpopulation of MNs (i.e., LMC neurons) at later developmental stages directs the formation or maintenance of LMC MNs.

**Figure 8—figure supplement 1.**
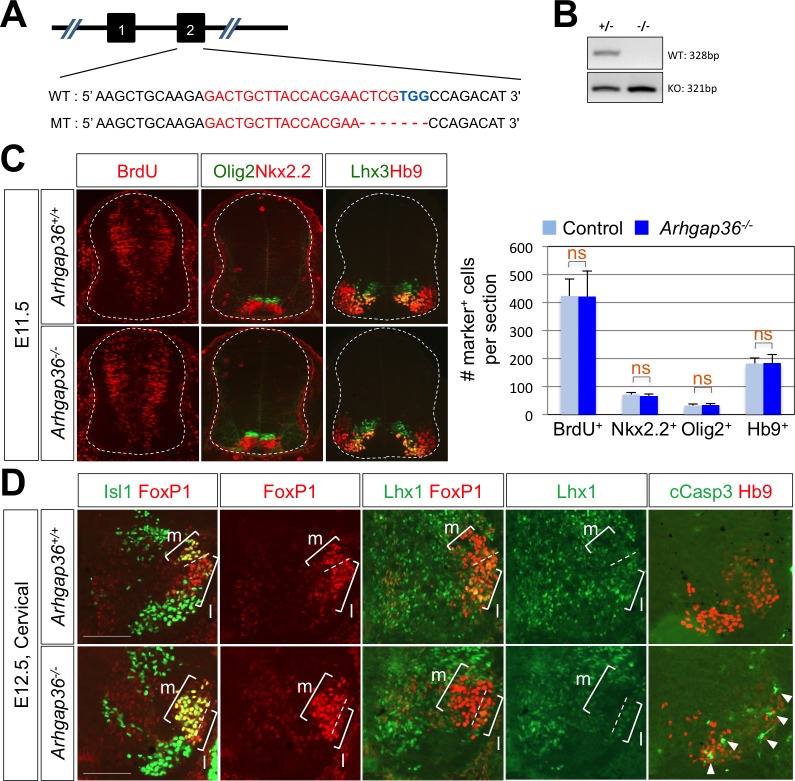
ARHGAP36 is required for proper LMC formation in mice. (**A**) Schematic showing the CRISPR/Cas9-mediated ARHGAP36 knockout mouse generation. Sequences for target exon2 of WT allele (upper) and ARHGAP36 KO allele (lower). The target sequence of sgRNA is marked in red, and the PAM sequence is in blue. (**B**) Genotyping PCR results show one DNA band for KO alleles from KO mouse genomic DNA and two DNA bands of WT and KO alleles from heterozygous. (**C**) IHC analyses of E11.5 *Arhgap36*^-/-^ mutant embryos (lower panels, n = 3) and control littermates (upper panels, n = 5). There is no defect in cell proliferation (BrdU), ventral patterning (Nkx2.2and Olig2) and motor neuron generation (Hb9) at early developmental stage. Data are mean ± s.d. ns, non-significant; n = 7 ~ 14 independent images per each sample. (**D**) IHC analyses of E12.5 *Arhgap36*^-/-^ mutant embryos (lower panels, n = 4) and control littermates (upper panels, n = 4). At cervical level, LMCm neurons are increased and LMCl neurons are decreased in *Arhgap36*^-/-^ mutant embryo. At the same time, there is an increase in apoptotic marker (cCasp3) in *Arhgap36*^-/-^ mutant spinal cord (arrowheads). Scale bars: 100 μm. 10.7554/eLife.46683.028Figure 8—figure supplement 1—source data 1.Source data for Figure 8—figure supplement 1C.

**Figure 8—figure supplement 2.**
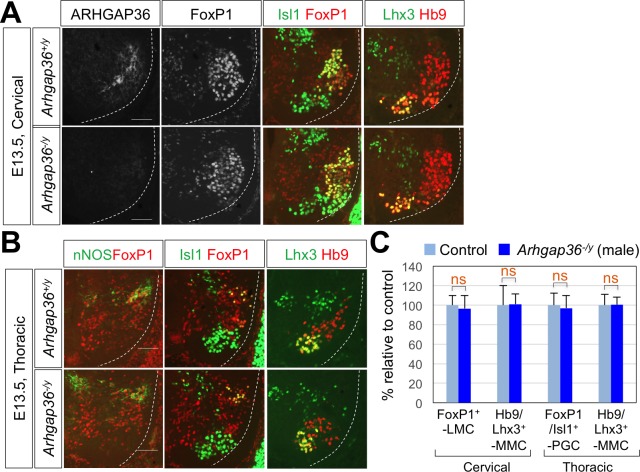
ARHGAP36 is not required for LMC formation in male mouse embryos. (**A**) IHC analyses of E13.5 *Arhgap36*^-/y^ mutant male embryos (n = 4) (lower panel) and their littermate controls (n = 5) (upper panel). At cervical level, the numbers of FoxP1^+^ LMC MNs and Hb9^+^/Lhx3^+^ MMC neurons did not change in *Arhgap36*^-/y^. (**B**) At thoracic level, there was no difference in nNOS^+^ PGC neurons, Hb9^+^/Isl1^+^ HMC neurons, and Hb9^+^/Lhx3^+^ MMC neurons compared to control littermates. Scale bars: 100 μm. (**C**) Quantification of the number of FoxP1^+^ LMC and Hb9^+^/Lhx3^+^ MMC at cervical level and FoxP1^+^/Isl1^+^PGC and Hb9^+^/Lhx3^+^ MMC at thoracic level in E13.5 male mouse embryonic spinal cord. Data are mean ±s.d. ns, non-significant; n = 6 ~ 12 independent images per each sample. 10.7554/eLife.46683.030Figure 8—figure supplement 2—source data 1.Source data for Figure 8—figure supplement 2C.

## Discussion

While the role of Shh signaling pathway to induce ventral progenitor domains in the spinal cord has been relatively well characterized (Persson et al., 2002; Jessell, 2000), its role in LMC specification has not been studied. In this report, we discovered a novel role of Shh in inducing LMC specification, which involves coordination of multiple signaling pathways by ARHGAP36, a key modulator of Shh signaling pathway. First, we discovered that Shh is expressed in MNs at brachial and lumbar levels of the spinal cord where LMC neurons are specified and is required for proper LMC formation in developing chick and mouse spinal cord (Figures 1–3). Second, we identified ARHGAP36, along with Shh, as a protein highly expressed in the LMC regions of differentiating MNs (Figure 5C). Third, ARHGAP36 modulates the activity of PKA, an inhibitor of Shh pathway, thereby enhancing the activity of Gli-dependent transcription in the spinal cord (Figure 6—figure supplement 1). Fourth, ARHGAP36 levels seem to be tightly regulated by AKT during MN generation, as shown by the increase of ARHGAP36 protein levels by WT AKT and a constitutively active form of AKT and a decrease in ARHGAP36 levels by a dominant negative form of AKT and AKT inhibitor (Figure 7 and Figure 7—figure supplements 1–2). Fifth, deletion of *Arhgap36* in mouse results in specific reduction of FoxP1^+^ LMC MNs in the developing mouse embryonic spinal cord (Figure 8), which is similar to what was observed in Shh knock-down chick spinal cord and *Shh*-cKO mouse spinal cord (Figures 2 and 3). Taken together, our results reveal a regulatory axis consisting of Shh-AKT-ARHGAP36-PKA, which plays crucial roles in modulating the activity of Shh signaling in a spatiotemporal manner for LMC specification.

Once MN progenitors, produced in the pMN progenitor domain of the ventral neural tube in response to the morphogen Shh, give birth to MNs (Jessell, 2000), MNs are further organized into distinct motor columns that are responsible for innervating each target muscle along the rostral-caudal neural tube (Dasen and Jessell, 2009; Stifani, 2014). MMC neurons innervate dorsal epaxial muscles, whereas HMC neurons project to the ventral hypaxial muscles. The LMC neurons innervate limb muscles and PGC neurons innervate sympathetic ganglia (Stifani, 2014; Dasen and Jessell, 2009). Motor column specific transcription factors and morphogenetic signaling molecules collaborate to define MN subtype specification (Shirasaki and Pfaff, 2002; Lee and Pfaff, 2001). RA is essential for the diversification of MN subtype and MN columnar organization. Also Hox genes, which encode a family of transcription factors, determine MN subtypes and there is a clear relationship between Hox protein expression and motor columnar specification. Moreover, FoxP1 has been shown to function as a critical Hox cofactor in regulating MN subtype diversity especially for specification of both the LMC and PGC neurons (Rousso et al., 2008; Pfaff, 2008; Dasen et al., 2008; Arber, 2008). It has been shown that abnormal expression of Hox proteins within postmitotic MNs result in subtype switching (Jung et al., 2010; Wu et al., 2008; Dasen et al., 2005; Lin and Carpenter, 2003). The spatiotemporal expression of these HD factors together with extrinsic signaling suggest that MN subtype identity remains plastic even after they exit the cell cycle and it should be tightly regulated to generate proper MN columnar subtypes. It is intriguing that two prominent extrinsic cues, RA and Shh, participate in LMC specification. Future studies should be directed at elucidating whether Shh pathway involving ARHGAP36 integrates with RA and Hox genes in LMC specification.

Because LMC and PGC neurons do not express Lhx3, it is not clear whether ARHGAP36 induced by the Isl1-Lhx3 complex at earlier stages of MN development (Figure 5) persists in LMC and PGC neurons or another mechanism independently induces the expression of ARHGAP36 in these specific motor columns at later stages. Because Shh stabilizes ARHGAP36 through AKT activation (Figure 7) and Shh is only expressed in LMC neurons at later developmental stages (Figure 1), Shh may be responsible for the continuous existence of ARHGAP36 at least in LMC neurons via its ability to stabilize ARHGAP36 protein. It is also possible that Hox and its cofactor Pbx may additionally upregulate the expression of ARHGAP36 at later stages of MN columnar specification (Hanley et al., 2016).

We found that there was no obvious defect in the general MN formation in *Arhgap36* knockout mouse embryonic spinal cord at early stages, suggesting that its function is not necessary for the initial MN differentiation. This conclusion is supported by the lack of Shh expression in early born MNs and with our notion that Shh agonist is likely needed for the activity of ARHGAP36 (Figure 6—figure supplement 2) as well as our finding that overexpression of AKT shows specific effect only on FoxP1^+^ LMC neurons (Figure 7). Because ARHGAP36 is also expressed in early born MNs, however, ARHGAP36 may also have a critical role in initial MN generation. This action of ARHGAP36 may have a redundant functional homologue, given no deficits were observed in early MN generation in *Arhgap36* knockout mouse embryonic spinal cord. Furthermore, there was no obvious defect even in the LMC formation in male mutant spinal cord at later stages. Based on the amino acid sequence of ARHGAP36, it is predicted to be a Rho GAP family member, but it lacks the ‘arginine finger’ motif for Rho GTPase activity (Rittinger et al., 1997), suggesting that the catalytic GAP domain is not required for Gli activation. *Arhgap6* is the closest homolog of *Arhgap36*, which does not affect Shh activation (Rack et al., 2014). It will be interesting to further investigate whether another functional homologue to ARHGAP36 controls initial MN generation as well as LMC formation particularly in male embryos. At E12.5, we observed that LMCm (Isl1^+^/FoxP1^+^) neurons are increased, whereas LMCl (Lhx1^+^/FoxP1^+^) neurons are decreased in *Arhgap36* mutant spinal cord (Figure 8—figure supplement 1). But later on, the number of FoxP1^+^ LMC neurons decreased significantly in *Arhgap36* deficient mouse embryonic spinal cord. These results suggest that the increased early born LMCm neurons in the *Arhgap36* mutant spinal cord somehow adopt other cell fates or degenerate. These possibilities need to be further investigated in the future. Later born LMCl neurons have to migrate through earlier born LMCs (Sockanathan et al., 2003; Maden, 2006) but it is not known whether early born LMCm neurons can change their fate into LMCl neurons by receiving signals such as RA and Shh from the neighboring cells. It will be interesting to further investigate whether early born LMCm neurons affect LMCl neuron specification through Shh signaling pathway and its modulator ARHGAP36.

In the developing central nervous system, a number of differentiated cells degenerate during development (Buss et al., 2006; Oppenheim, 1991). Initially overproduced MNs undergo cell death by competing for a limited amount of neurotropic factors when they arrive at the target muscles (Yamamoto and Henderson, 1999). LMC neuronal axons navigate through a very long distance to their final destinations by integrating intrinsic factors and extrinsic pro- and anti-apoptotic signals. Based on the well-known function of AKT in cell survival, we speculate that ARHGAP36 proteins stabilized by AKT may also function to maintain the proper number of differentiated LMC neurons by blocking their apoptosis. Indeed, we observed an increase in cell death with a reduction of FoxP1^+^ LMC neurons in *Arhgap36* KO mouse (Figure 8—figure supplement 1D), suggesting that ARHGAP36 may function as an essential downstream effector of AKT-directed cell survival.

It remains to be further investigated how ARHGAP36 affects the FoxP1^+^ LMC cell fate specification. It has been shown that FoxP1 expression is regulated by PI3K/AKT/p70S6K signaling cascade in breast cancer (Halacli and Dogan, 2015). These results further support our proposal that the expression of FoxP1 in LMC neurons is likely regulated by activated AKT in collaboration with ARHGAP36. Furthermore, ARHGAP36 is up-regulated in medulloblastoma where Shh pathway is aberrantly activated (Rack et al., 2014), suggesting that similar mechanisms may be applied to tumorigenesis. Therefore, it will be interesting to further study the function of ARHGAP36 in Shh-dependent tumorigenesis, for instance using the cerebellar granule neuron progenitors (GNPs) from *Arhgap36*^-/-^ mice.

## Materials and methods

**Key resources table:** 

Reagent type (species) or resource	Designation	Source or reference	Identifiers	Additional information
Genetic reagent (*M. musculus*)	*Shh^f/f^*	Jackson Laboratories	RRID:MGI:2165468	
Genetic reagent (*M. musculus*)	*Olig2-Cre*	PMID: 18046410		Dr. Bennett G. Novitch (University of California, Los Angeles)
Genetic reagent (*M. musculus*)	*Arhgap36^-/-^*	This paper		Exon2 targetting sgRNA made by ToolGEN, injected by KRIBB
Transfected construct (*M. musculus*)	*Arhgap36*-(enhancer)^2^:LUC	This paper		Arhgap36 enhancer containing HxRE sequence cloned into TK-LUC vector
Transfected construct (*M. musculus*)	*Arhgap36*-(enhancer)^2^:GFP	This paper		Arhgap36 enhancer containing HxRE sequence cloned into TATA-GFP vector
Transfected construct (*R. norvegicus*)	*Isl1*	PMID: 22343290		
Transfected construct (*M. musculus*)	*Lhx3*	PMID: 22343290		
Transfected construct (*E. coli*)	*β-galactosidase*	PMID: 22343290		
Transfected construct (*M. musculus*)	Arhgap36	Open Biosystems	Accession: BC145645	
Transfected construct (*M. musculus*)	PKA WT	PMID: 23644383		
Transfected construct (*M. musculus*)	PKA K73H	PMID: 23644383		
Transfected construct (*M. musculus*)	AKT2 CA	Addgene	RRID:Addgene_9016	myristoylated form
Transfected construct (*M. musculus*)	AKT2 DN	Addgene	RRID:Addgene_60128	
Sequence-based reagent	Shh shRNA_sense strand	This paper	PCR primers	GAT CCA AGC TCT TCT ACG TCA TCG TTC AAG AGA CGA TGA CGT AGA AGA GCT TTT TTT A
Sequence-based reagent	Shh shRNA_antisense strand	This paper	PCR primers	AGC TTA AAA AAA GCT CTT CTA CGT CAT CGT CTC TTG AAC GAT GAC GTA GAA GAG CTT G
Sequence-based reagent	Arhgap36 enhancer_F	This paper	PCR primers	ACTGCCTATTCGCATCGGCCTTTGA, for cloning
Sequence-based reagent	Arhgap36 enhancer_R	This paper	PCR primers	TTCTGCGGAGCCATTAGTGCGATTG, for cloning
Sequence-based reagent	mouse Arhgap36_F	This paper	PCR primers	TGG GAT CCA AGA GGA AGA TG, for RT-PCR
Sequence-based reagent	mouse Arhgap36_R	This paper	PCR primers	CAG CCA CAT CAT GGA CAT TC, for RT-PCR
Sequence-based reagent	mouse Cyclophilin A_F	This paper	PCR primers	GTC TCC TTC GAG CTG TTT GC, for RT-PCR
Sequence-based reagent	mouse Cyclophilin A_R	This paper	PCR primers	GAT GCC AGG ACC TGT ATG CT, for RT-PCR
Sequence-based reagent	mouse Arhgap36 enhancer_F	This paper	PCR primers	ACC TTG TAG CAG GAC TGG GGT, for ChIP
Sequence-based reagent	mouse Arhgap36 enhancer_R	This paper	PCR primers	AGC CAT TAG TGC GAT TGC TCT, for ChIP
Sequence-based reagent	Untr6_F	PMID: 18854042	PCR primers	TCA GGC ATG AAC CAC CAT AC, for ChIP
Sequence-based reagent	Untr6_R	PMID: 18854042	PCR primers	AAC ATC CAC ACG TCC AGT GA, for ChIP
Antibody	anti-Hb9/MNR2 (Mouse)	DSHB	DSHB Cat# 81.5C10, RRID:AB_2145209	IHC, 1:500
Antibody	anti-Isl1 (Rabbit monoclonal)	Abcam	Abcam Cat# ab109517, RRID:AB_10866454	IHC, 1:2000
Antibody	anti-FoxP1 (Rabbit polyclonal)	Abcam	Abcam Cat# ab16645, RRID:AB_732428	IHC, 1:1000
Antibody	anti-Nkx2.2 (Mouse monoclonal)	DSHB	DSHB Cat# 74.5A5, RRID:AB_531794	IHC, 1:100
Antibody	anti-Pax6 (Mouse monoclonal)	DSHB	DSHB Cat# pax6, RRID:AB_528427	IHC, 1:500
Antibody	anti-Olig2 (Rabbit polyclonal)	Abcam	Millipore Cat# AB15328, RRID:AB_2299035	IHC, 1:1000
Antibody	anti-β-gal (Chicken polyclonal)	Abcam	Abcam Cat# ab9361, RRID:AB_307210	IHC, 1:5000
Antibody	anti-Lhx3 (Rabbit polyclonal)	Abcam	Abcam Cat# ab14555, RRID:AB_301332	IHC, 1:500
Antibody	anti-nNOS (Rabbit polyclonal)	Immunostar	ImmunoStar Cat# 24287, RRID:AB_572256	IHC, 1:1000
Antibody	anti-Chx10 (Guinea pig polyclonal)	PMID: 18539116		IHC, 1:1000
Antibody	anti-GFP (Rabbit polyclonal)	Life Technologies	Thermo Fisher Scientific Cat# A-11122, RRID:AB_221569	IHC, 1:1000
Antibody	anti-Hb9 (Guinea pig polyclonal)	PMID: 30177510		IHC, 1:1000 rat Hb9 C-terminus (234–403 aa
Antibody	anti-ARHGAP36 (Rabbit polyclonal)	This paper		IHC, 1:2000 mouse ARHGAP36(201–590 aa)
Antibody	anti-HA (Mouse monoclonal)	Covance	Covance Research Products Inc Cat# MMS-101R-500, RRID:AB_10063630	IP, IB, 1:5000
Antibody	anti-Gli3 (Goat polyclonal)	R and D Systems	R and D Systems Cat# AF3690, RRID:AB_2232499	IB, 1:250
Antibody	anti-ARHGAP36 (Rabbit polyclonal)	Sigma-Aldrich	Sigma-Aldrich Cat# HPA002064, RRID:AB_1078891	IB, 1:2000
Antibody	anti-β-tubulin (Rabbit polyclonal)	Santa Cruz	Santa Cruz Biotechnology Cat# sc-9104, RRID:AB_2241191	IB, 1:2000
Antibody	anti-pSER	Cell Signaling	Cat. #9651	IB, 1:5000
Antibody	anti-TuJ1 (Mouse monoclonal)	Covance	Covance Research Products Inc Cat# MMS-435P, RRID:AB_2313773	IB, 1:5000 IHC, 1:5000
Antibody	anti-FoxP1 (Rabbit polyclonal)	abcam	Abcam Cat# ab16645, RRID:AB_732428	IB, 1:1000
Antibody	anti-pCREB (Rabbit monoclonal)	Cell Signaling	Cell Signaling Technology Cat# 9198, RRID:AB_2561044	IB, 1:1000
Cell line (*M. musculus*)	P19	ATCC	ATCC Cat# CRL-1825, RRID:CVCL_2153	embryonic carcinoma cells
Cell line (*Homo-sapiens*)	HEK293T	ATCC	ATCC Cat# CRL-3216, RRID:CVCL_0063	
Cell line (*M. musculus*)	A172L ESC	PMID: 22343290, 22039605		
Chemical compound, drug	iAKT1/2	Sigma Aldrich	A6730	10 μM
Chemical compound, drug	SAG	Calbiochem	MER-566660	0.25 μM
Chemical compound, drug	Lipofectamine 2000	Invitrogen	Cat. 52887	
Chemical compound, drug	Superfect	Qiagen	Cat. 301307	
Chemical compound, drug	SuperScript III First-Strand Synthesis System	Invitrogen	Cat. 18080085	
Chemical compound, drug	SYBR-Green kit	Enzynomics	RT501S	
Software, algorithm	GPS 3.0	PMID: 15980451		http://gps.biocuckoo.cn/

### DNA constructs

*Arhgap36-*(enhancer)^2^:LUC and *Arhgap36-*(enhancer)^2^:GFP reporters were constructed with two copies of enhancer genomic fragments (268 bp) into synthetic TK-LUC or TATA-GFP vectors; primers used for enhancer genomic PCR are forward: 5’-ACT GCC TAT TCG CAT CGG CCT TTG A-3’ and reverse: 5’-TTC TGC GGA GCC ATT AGT GCG ATT G-3’. Rat *Isl1* and mouse *Lhx3* and *LacZ* genes were cloned in pCS2 containing a HA, Flag or myc-epitope tag for expression in mammalian cells and chick embryos, as previously described (Lee et al., 2012; Lee et al., 2004; Lee and Pfaff, 2003; Thaler et al., 2002). Mouse *Arhgap36* gene was purchased from Open Biosystems and cloned into HA or Flag-tagged pCS2 vectors and UAS enhancer-CMVmini promoter containing vector. Hb9 promoter region (1212 bp) was cloned to pCS2 vector containing Gal4 transcriptional activator gene. PKA WT and K73H mutant were cloned into HA-pCS2 vector. HA-tagged AKT2 WT, dominant negative form (Addgene #60128) and constitutively active form AKT2 (Addgene #9016) were cloned into pcDNA3 or pCS2 vector. Short hairpin RNA (shRNA) of chick Shh was cloned into EFU6-300 vector; sense strand: 5’-GAT CCA AGC TCT TCT ACG TCA TCG TTC AAG AGA CGA TGA CGT AGA AGA GCT TTT TTT A-3’, antisense strand: 5’-AGC TTA AAA AAA GCT CTT CTA CGT CAT CGT CTC TTG AAC GAT GAC GTA GAA GAG CTT G-3’.

### Chick *in ovo* electroporation, Immunohistochemistry and in situ hybridization assays

DNAs were injected into the lumen of the neural tube of HH stage 13 chick embryos, which were then electroporated (Thaler et al., 2002). The embryos were harvested 3 or 4 days post-electroporation and fixed in 4% paraformaldehyde, embedded in OCT and cryosectioned in 12 μm thickness for IHC assays or 18 μm thickness for ISH with digoxigenin-labeled probes. Each set of chick electroporation experiments was repeated independently at least three times. Representative sets of images from reproducible results were presented.

For IHC assays, the following antibodies were used; mouse anti-Hb9/MNR2 (DSHB, 5C10, 1:500), rabbit anti-Isl1 (abcam, ab109517, 1:2000), rabbit anti-FoxP1 (abcam, ab16645, 1:1000), mouse anti-Nkx2.2 (DSHB, 5A5, 1:100), mouse anti-Pax6 (DSHB, 1:500), rabbit anti-Olig2 (abcam, ab15328, 1:1000), chicken anti-β-gal (Abcam, ab9361, 1:5000), rabbit anti-Lhx3 (abcam, ab14555, 1:500), rabbit anti-nNOS (Immunostar, 1:1000), guinea pig anti-Chx10 (1:1000) (Lee et al., 2008; Thaler et al., 1999) and rabbit anti-GFP (Life Technologies, A11122, 1:1000). Guinea pig anti-Hb9 antibody was raised from guinea pig using rat Hb9 C-terminus (234–403 aa) as an antigen and rabbit anti-ARHGAP36 antibody was raised from rabbit with mouse ARHGAP36 (201–590 aa) as an antigen.

For ISH analyses, mouse *Arhgap36, Akt1, Akt2, Akt3, Prkaca, Prkacb, Prkar1a, Prkar1b, Prkar2a, Prkar2b* chick *Ptch1, Gli1,* and *Shh* were cloned to pBluescript vector and used to generate digoxigenin-labeled riboprobes.

### Mice

All mouse works were performed under an approved protocol (SNU-150123-1-2) by the Institutional Animal Care and Use Committee (IACUC) at Seoul National University. *Arhgap36* mice were generated using CRISPR/Cas9 system. We injected Cas9 mRNA and single guide RNA (sgRNA) directly into mouse embryos to target exon 2 of *Arhgap36* gene and obtained a mouse line with seven nucleotides deleted (Figure 8—figure supplement 1). This resulted in a premature stop codon producing a truncated ARHGAP36 protein of 84 amino acids. Targeting sgRNA is 5’-GAC TGC TTA CCA CGA ACT CGT GG-3’, (ToolGEN). Cas9 mRNA and sgRNA mixtures were injected into one-cell embryos (Korea Research Institute of Bioscience and Biotechnology). *Arhgap36^-/y^* male mice were crossed with *Arhgap36^+/-^* female mice to get KO mutant embryos for the analyses. *Shh^f/f^* and *Olig2-Cre* mouse lines were described previously (Lewis et al., 2001; Dessaud et al., 2007). *Shh^f/f^* mice were obtained from the Jackon Laboratories (Bar Harbor, ME). Mouse embryos were collected at indicated developmental stages and processed similarly to chick embryos as described above.

### Luciferase reporter assays

P19 embryonic carcinoma cells (ATCC, #CRL-1825) were cultured in MEM supplemented with 10% fetal bovine serum (FBS). For luciferase assays, P19 cells were plated in 48-well plate and incubated for 24 hr, followed by transient transfections using Lipofectamine 2000 (Invitrogen). An actin-β-galactosidase plasmid was co-transfected for normalization of transfection efficiency and empty vectors were used to equalize the total amount of DNA. Cells were harvested 36–40 hr after transfection. Cell extracts were assayed for luciferase activity and the values were normalized with β-galactosidase activity. All transfections were repeated independently at least three times. Data are presented as the mean of triplicate values obtained from representative experiments. Error bars represent standard deviation.

### Co-immunoprecipitation and immunoblotting assays

HEK293T cells (ATCC, #CRL-3216) were cultured in DMEM media supplemented with 10% FBS. For co-immunoprecipitation, HEK293T cells were seeded on 10 cm tissue culture dishes, cultured in DMEM media supplemented with 10% FBS, and transfected with the expression vectors tagged with Flag, and HA using Superfect (Qiagen) or calcium phosphate. Cells were treated with AKT1/2 kinase inhibitor, iAKT1/2 (Sigma Aldrich, A6730) at 10 μM for 20 hr and then cells were harvested and lysed in IP buffer (20 mM Tris-HCl, pH 8.0, 0.5 % NP-40, 1 mM EDTA, 150 mM NaCl, 2 mM PMSF, 10% Glycerol, 4 mM Na3VO4, 200 mM NaF, 20 mM Na-pyroPO4, and protease inhibitor cocktail). In these studies, immunoprecipitations were performed with mouse anti-HA antibody (Covance). Immunoblotting assays were performed using goat anti-Gli3 (R and D Systems, AF3690, 1:250), rabbit anti-ARHGAP36 (Sigma, HPA-002064, 1:2000), mouse anti-HA (Covance, 1:5000), rabbit anti-β-tubulin (Santa Cruz, sc-9104, 1:2000), rabbit anti-pSER (Cell Signaling, #9651, 1:5000), mouse anti-TuJ1 (Covance, 1:5000), rabbit anti-FoxP1 (abcam, ab16645, 1:1000) and anti-pCREB (Cell Signaling, 9198S, 1:1000) antibodies.

### Differentiation of ESC lines, RNA extraction and Quantitative RT-PCR

The mouse A172L ESC line (Iacovino et al., 2011; Lee et al., 2012) was maintained in an undifferentiated state on 0.1% gelatin-coated dishes in the ESC growth media that consist of knockout DMEM, 10% FBS, 0.1 mM nonessential amino acids, 2 mM L-glutamine, 0.1 mM β-mercaptoethanol, and recombinant leukemia inhibitory factor (LIF) (1000 units/ml, Chemicon). Cell line was tested periodically for *Mycoplasma* contamination and has not shown evidence of *Mycoplasma*. For MN differentiation assays, ESCs were trypsinized and plated in ADFNK media containing advanced DMEM with F-12:neurobasal medium, 200 mM L-glutamine, 1% penicillin/streptomycin, 10% knockout serum replacement, and 0.1% β-mercaptoethanol in suspension as cell aggregates for 2 days. The ESC aggregates (embryoid bodies, EBs) were treated with all-trans RA (1 μM) and Shh agonist, SAG (0.25 μM, Calbiochem) for 2 days. Then, RA and SAG-treated EBs were cultured without or with AKT1/2 inhibitor, iAKT1/2 (10 μM, Sigma Aldrich) in the presence of RA and SAG for another 2 days. At Day6, cells were harvested for RNA extraction, IHC and immunoblotting assays. Total RNAs were extracted using the Trizol (Invitrogen) and reverse-transcribed using the SuperScript III First-Strand Synthesis System (Invitrogen). The levels of mRNAs were determined with quantitative RT-PCR using SYBR-Green kit (Enzynomics) and CFX Connect Real-Time PCR detection system (Biorad). The following primers were used; mouse *Arhgap36*, 5’-TGG GAT CCA AGA GGA AGA TG, 5’-CAG CCA CAT CAT GGA CAT TC, and *Cyclophilin A,* 5’-GTC TCC TTC GAG CTG TTT GC, 5’-GAT GCC AGG ACC TGT ATG CT. Data are shown as the mean of duplicate values obtained from representative experiments. Error bars represent standard deviation.

### ChIP assays

ChIP was performed in mouse embryonic spinal cords as described previously (Nam and Lee, 2016; Cho et al., 2014; Lee et al., 2013). The spinal cords were microdissected from E12.5 mouse embryos and cells were dissociated and subjected to ChIP assays. Cells were washed with PBS buffer, fixed in 1% formaldehyde for 10 min at room temperature, and quenched by 125 mM glycine. Cells were washed with Buffer I (0.25% Triton X-100, 10 mM EDTA, 0.5 mM EGTA, 10 mM Hepes, pH 6.5) and Buffer II (200 mM NaCl, 1 mM EDTA, 0.5 mM EGTA, 10 mM Hepes, pH 6.5) sequentially. Then, cells were lysed with lysis buffer (0.5% SDS, 5 mM EDTA, 50 mM Tris·HCl, pH 8.0, protease inhibitor mixture) and were subjected to sonication for DNA shearing. Next, cell lysates were diluted 1:10 in ChIP buffer (0.5% Triton X-100, 2 mM EDTA, 100 mM NaCl, 50 mM Tris·HCl, pH 8.0, protease inhibitor mixture) and, for immunoclearing, were incubated with IgG and protein A agarose beads for 1 hr at 4°C. The supernatant was collected after quick spin and incubated with anti- IgG (Santa Cruz), anti-Isl1 (Tsuchida et al., 1994) and anti-Lhx3 (Sharma et al., 1998) antibodies and protein A agarose beads to precipitate the Isl1-Lhx3 complex/chromatin complex for overnight at 4°C. The purified final DNA samples were subjected to quantitative PCR reactions. The primers that were used for ChIP-PCR are; *Arhgap36* enhancer, forward, 5’-ACC TTG TAG CAG GAC TGG GGT, reverse, 5’-AGC CAT TAG TGC GAT TGC TCT, and *Untr6*, forward, 5’-TCA GGC ATG AAC CAC CAT AC, reverse, 5’-AAC ATC CAC ACG TCC AGT GA.

## Data Availability

All data generated or analysed during this study are included in the manuscript and supporting files. The following previously published datasets were used: LeeSShenRChoHHKwonRJSeoSYLeeJWLeeSK2013STAT3 promotes motor neuron differentiation by collaborating with motor neuron-specific LIM complexNCBI Gene Expression OmnibusGSE5099310.1073/pnas.1302676110PMC371078723798382 MazzoniEOMahonySClosserMMorrisonCANedelecSWilliamsDJAnDGiffordDKWichterleH2013Synergistic binding of transcription factors to cell-specific enhancers programs motor neuron identity.NCBI Gene Expression OmnibusGSE3145610.1038/nn.3467PMC382049823872598

## Additional files

Transparent reporting form
