# Supplementary material for: Critical roles of ARHGAP36 as a signal transduction mediator of Shh pathway in lateral motor columnar specification
Source: eLife. 2019 Jul 15;8:e46683. doi: 10.7554/eLife.46683 (PMC6658197; doi:10.7554/eLife.46683)
Supplement: Peer review history

# Decision letter

**DOI:** 10.7554/eLife.46683.038

**Affiliations:** The Francis Crick InstituteUnited Kingdom

In the interests of transparency, eLife includes the editorial decision letter and accompanying author responses. A lightly edited version of the letter sent to the authors after peer review is shown, indicating the most substantive concerns; minor comments are not usually included.

[Editors’ note: this article was originally rejected after discussions between the reviewers, but the authors were invited to resubmit after an appeal against the decision.]

Thank you for submitting your article " Critical roles of ARHGAP36 as a signal transduction mediator of Shh pathway in lateral motor columnar specification" for consideration by *eLife*. Your article has been reviewed by two peer reviewers, one of whom is a member of our Board of Reviewing Editors, and the evaluation has been overseen by Marianne Bronner as the Senior Editor. The reviewers have discussed the reviews with one another and the Reviewing Editor has drafted this decision.

Summary:

This manuscript addresses the role of Sonic hedgehog expressed by postmitotic spinal neurons in chick and mouse embryos. The paper shows that Shh is required for the formation of the lateral motor column at brachial level and that it acts by activating AKT signaling, which stabilises the ARHGAP36 protein, induced in motorneurons by Isl1-Lhx3 complexes. ARHGAP36 in turn inhibits the kinase activity of PKA, which dephosphorylates Gli proteins and favours the formation of activating over repressing forms of Gli proteins.

Comments:

The reviewers found this study novel and interesting. However, reviewer 2 was concerned that electroporation experiments in chick embryos did not rule out an effect of the manipulation on patterning or proliferation of progenitor cells rather than a direct effect on postmitotic motorneurons, because electroporations were performed at a stage when patterning was not fully established and the expression constructs did not contain a motorneuron-specific promoter (reviewer 2, points 7 and 19). Similarly, the reviewer was concerned that Shh deletion in mouse embryos using the Olig2-Cre deleter line did not exclude patterning or proliferation defects due to elimination of Shh in ventral progenitors rather than in postmitotic neurons (reviewer 2 point 11). After consultation between the reviewer, reviewer 1 agrees with these points. As Olig2 is initially expressed in the ventral-most domain of the spinal cord except in the most medial cells (e.g. Dessaud et al., 2007), it cannot be excluded that Shh has been deleted by Olig2-Cre from cells in the early Olig2+ progenitor domain, resulting in the observed phenotype.

Other concerns include the lack of sufficient analysis to exclude patterning, proliferation or survival defects in the manipulated chick and mouse embryos (reviewer point 1; reviewer 2 points 5 and 6), and the insufficient quantification of the data (reviewer 1 point 2; reviewer 2 points 4, 8, 14, 17, 19, 22-25).

Addressing these different concerns would require new experiments, which would likely take substantially longer than the two months normally allocated to the revision of *eLife* manuscripts. We have therefore regretfully decided to reject your manuscript.

*Reviewer #1:*

This paper reports a novel role of Shh, produced by postmitotic motor neurons, in the specification of the lateral motor column (LMC) in the chick and mouse spinal cord. The authors show that AKT stabilises the Rho GAP protein ARHGAP36 in the LMC region, and that ARHGAP36 in turn inhibits the activity of PKA and thereby enhances the production of activating forms of Gli proteins.

The identification of a role for Shh in motor neuron specification and the elucidation of the downstream pathway involving a novel regulator and multiple signaling molecules is of significant interest. The data supports the conclusions of the authors and the work is of excellent quality and well presented. I have a few requests to improve the data, the analysis and discussion to strengthen the conclusions of the paper.

1) Shh acts as a mitogen in many systems but the authors rule out that it is the case here by labelling MN progenitors with antibodies to Olig2 and *Sox2* in Figure 2C. However the Shh LOF phenotype in Figure 2A only affects the generation of a small minority of MNs, which could be due to a proliferation defect affecting only a small fraction of progenitors which may not be picked up by the current analysis. To rule this out, the authors should examine proliferating cells more directly, eg with a short pulse of EdU. This should detect a putative proliferation defect with more sensitivity than examining total progenitor numbers.

2) The ectopic expression of ARHGAP36 in the ventral spinal cord in Figure 5B increases visibly the number of FoxP1+ LMC neurons while Lhx3+ Hb9+ MMCm neurons do not appear affected. However to conclude that ARHGAP36 induces LMC neurons rather than converting MMCm into LMC, requires quantification of the data.

3) The conclusion of the experiment in Figure 6A that AKT stabilises ARHGAP36 protein should be strengthened by measuring ARHGAP36 half-life in HEK293T cells in the presence and absence of AKT, and also by showing that manipulating AKT activity does not affect ARHGAP36 transcription.

4) The regulation of ARHGAP36 expression may need further discussion. Figure 6 shows that AKT controls ARHGAP36 protein stability, but how its transcription is regulated is less clear. Figure 2 S1, shows a reduction of its expression in Shh cKO mice. How does Shh regulates its expression and are there other signals involved? Along the same line, the arrow between Shh and P-AKT in Figure 7D may need more explanation.

*Reviewer #2:*

The manuscript by Nam and cols, entitled "Critical roles of ARHGAP36 as a signal transduction mediator of Shh pathway in lateral motor columnar specification" proposes a novel role for Shh signalling in spinal cord development.

A putative role for Shh activity as a signal instructing the selection of specific MNs subpopulations is novel and attractive. It is based in the observation of Shh expression in postmitotic MNs, which is not fully novel, but has not been well characterized previously.

Authors also find that the Rho GAP family member *Arhgap36* is expressed in postmitotic MNs, and propose that it might act as a Shh effector in MN subpopulations selection. In different cell contexts, *Arhgap36* has previously been shown to inhibit PKA activity, hence regulating Shh signalling. However, it would be nice to fully define its in vivo relevance, in the context of selection of specific MNs subpopulations. In summary, the manuscript contains some novel and interesting data, however it lacks definitive demonstration of some of the conclusions raised by the authors.

Hence, I believe that, in the present format, the manuscript has important gaps that need to be fulfilled.

Specific points:

In order to propose a new role for Shh signalling as an instructive signal the selection of specific MNs subpopulations, I believe that authors should improve the characterization of the MN subpopulations expressing Shh.

1) In the chick embryo spinal cord, Shh expression has previously been reported in postmitotic motor neurons of the LMC (at stage HH15), extending later to the MMC (Stage HH35) (Figure 1 in Oppenheim et al., 1999). However, Nam and cols, in this manuscript shows Shh expression restricted to LMC neurons at the brachial level. The selected section showed in Figure 1A shows Shh expression restricted to the mLMC (and excluded from lLMC), as well as excluded from MMC. Could authors clarify these contradictory issues?

2) Panel B of this figure (expression in mouse embryos) do not show this restricted expression to mLMC. Are these specie specific differences? Or developmental stage differences?

3) A detailed temporo-spatial expression of Shh in postmitotic MNs should be provided. These should be easy to clarify combining with the available and well defined markers for each MN column (see for example Adams et al., 2015)

In order to analyse the role of Shh in MN subtype specification; loss-of-function experiments in chick embryo NT were performed by electroporation at HH stage 13 chick embryos (Materials and methods), at which patterning of the NT is not definitely established (see for example Cayuso et al., 2006).

4) Controls showing quantification of Shh- loss are required, particularly since defects appeared to be mild, despite the length of the experiments (96 hours post-electroporation at HH13). For example, Gli-bs-luc experiments to show the efficiency of the Shh-LOF vector.

5) Moreover, the role of Shh signalling in proliferation and survival of neural progenitors are also well stablished, hence controls ruling out these defects are required.

6) Controls showing markers for progenitors should be included in order to rule out patterning defects.

7) I strongly believe that, in order to analyse a specific role for Shh signalling in postmitotic MN subtype generation, experiments should have been done using a MN specific driver (for example the well characterized HB9) to avoid patterning and proliferation defects.

8) Quantitative data indicating the total number of MNs, versus sub-type specific MNs should be provided. It is important to understand whether LMNs have switch their fate, of whether LMNs are not being generated.

9) Quantitative data in Figure 1A panel D shows FoxP1+ cells, are these islet1+? Or islet1-? Same for Hb9, these analyses will discriminate between lLMB vs mLMC. Quantitative data in Figure 1A panel E shows Lhx3+ cells, same for the combination with additional marker to restrict the phenotype to specific MN subtype

10) A schematic drawing of the MN columns, including markers and phenotype would be of great help.

11) The same holds for the analysis of the Shh-Olig2C mutant phenotype. These experiments should have been done using a MN specific driver (for example the well characterized HB9CRE line, available at Jackson) to avoid patterning and proliferation defects.

12) Please indicate the total number of MNs, versus sub-type specific MNs. Please indicate whether FoxP1+ cells are islet1 and Hb9 +/-.

To identify the candidate effector genes, authors searched for target genes of the Isl1-Lhx3 transcriptional complex, well characterized previously by this group. What is the link between this searching strategy and Shh signalling, it is not clear to me, but they identify the Rho GAP family member *Arhgap36*, which is known to activate Gli transcriptional responses through the inhibition of PKA activities.

13) It would be nice to see, among the many genes regulated by the Isl1-Lhx3 transcriptional complex, whether there might be a cluster of HH-signalling components?

14) Authors identify a HxRE of the *Arhgap36* gene, and test the in vivo activity by electroporation experiments in chick embryo NT. Please provide quantitative data indicating number of embryos analysed. Provide quantitative data indicating HB9 ectopic cells induced. Please provide electroporation time course, as well as negative control experiments.

15) I believe that the data provided here demonstrate that the Isl1-Lhx3 complex is sufficient to induce *Arhgap36* expression, whether this induction is direct is not fully demonstrated.

Next authors analyse *Arhgap36* expression in postmitotic neurons. This is an important issue, since Shh might be acting either in a paracrine manner, or in a cell autonomous manner, to instruct MNs subtype identity.

16) Please provide a better characterization of *Arhgap36* in MN subtypes (co-localization with MN subtype markers) and co-localization (or not) with Shh, with cellular resolution.

17) Please provide *Arhgap36* expression data in chick embryos MNs. It would be interesting to see the conservation of it expression in MNs.

Next authors test whether the Shh-pathway is activated upon over-expression of *Arhgap36*. Experiments in chick embryo NT were performed by electroporation of ARHGAP36 at HH stage 13 chick embryos (Materials and methods). These gain-of-function experiments produce NT overgrowth, corresponding to the over-activation of Shh signalling, in addition to ventralization of the entire NT. Quantitative data indicating number of embryos analysed is missing, electroporation time course, as well as quantification of marker-specific ectopic cells should be provided.

18) I believe that these experiments are controls of the capacity of ARHGAP36 in activating Shh-mediated responses in vivo. Since this has been previously documented in other systems, these experiments can go as supplementary information.

19) Authors claim that over-expression of ARHGAP36 in ventral NT, harvested at 4 dpe the number of FoxP1+ LMC neurons increased drastically, suggest that ARHGAP36 is sufficient to direct LMC fate-determination. Again here, I believe that these experiments should have been done using a MN HB9 specific driver to avoid patterning and proliferation defects. With the present experimental design, it is very difficult to assign a direct role for ARHGAP36 in postmitotic MN subpopulations selection, other that the role in MN progenitors

Again in these experiments, quantitative data indicating number of embryos analysed is required, electroporation time course is missing. Quantification of marker-specific ectopic cells should be provided.

Next, authors show that PKA activity is inhibited by ARHGAP36. However, this observation is not novel, since Eccles et al. (2016) showed that ARHGAP36 combines two distinct inhibitory mechanisms to antagonise PKA signalling; it blocks PKA catalytic activity and it targets PKAc for ubiquitin-mediated degradation, resulting in activation of the Shh pathway.

20) I don't see the point in analysing other targets but PKA in the context of MN specification, hence I believe that panels C,D,E from Figure 5 are not relevant in this context.

21) Again, in the context of Shh/ ARHGAP36 signalling in MN subtype selection, I do not see the relevance of the AKT experiments shown if Figure 6. I believe that should be removed.

Moreover, Zhang et al. (2019) find that Patched1 interacts with ArhGAP36 to the centrosome and stabilizes the PKA negative regulator (this paper should be quoted in the manuscript).

Zhang et al., Patched1-ArhGAP36-PKA-Inversin axis determines the ciliary translocation of Smoothened for Sonic Hedgehog pathway activation. Proc Natl Acad Sci U S A. 2019 Jan 15;116(3):874-879

22) Authors show reduced expression of ARHGAP36 in Shh-KO mice (Figure S4), again without any quantitative data.

Finally, authors generate an ARHGAP36 mutant mice. Authors claim that at early developmental stages, there was no defect in MN generation. Quantitative data on this control observation should be provided.

23) Analysis of the phenotype generated by ARHGAP36_CRISPR mutant in MNs at the brachial and the thoracic levels of the developing spinal cord, again require quantitative data indicating that the total number of MNs, have not changed at thoracic levels.

24) Quantitative data indicating that the total number of MMC and the total number of V2 interneurons, have not changed at brachial levels.

25) Quantitative data indicating the total MN numbers versus sub-type specific MNs should be provided. It is important to understand whether, in the absence of ARHGAP36, LMNs have switch their fate, of whether LMNs are not being generated.

# Reply: Author response

**DOI:** 10.7554/eLife.46683.039

[…] Addressing these different concerns would require new experiments, which would likely take substantially longer than the two months normally allocated to the revision of eLife manuscripts. We have therefore regretfully decided to reject your manuscript.

One of the main concerns of reviewers was if Shh deletion in mouse embryos using the Olig2-Cre deleter line could exclude patterning or proliferation defects due to the elimination of Shh in ventral progenitors rather than in postmitotic neurons. The reviewer pointed out that "As Olig2 is initially expressed in the ventral-most domain of the spinal cord except in the most medial cells [1], it cannot be excluded that Shh has been deleted by Olig2-Cre from cells in the early Olig2^+^ progenitor domain, resulting in the observed phenotype."

We understand why the reviewer expressed the concern as Shh secreted from the floor plate is critical for the patterning of the ventral neural tube, thus affecting the subsequent motor neuron differentiation. However, the data from my lab and other groups indicate that the expression of Shh in the floor plate is not compromised in our Shh-cKO;Olig2-Cre mouse model, excluding the possibility that the observed phenotype is caused by Shh deletion in the floor plate.

First, it is important to note that Shh is specifically expressed in the floor plate, but not in neurons, at the early stage of spinal cord development when the ventral progenitors undergo Shh-dependent patterning. Our in situ hybridization data (Figure 1A,B) are consistent with the previous reports. Further supporting the floor plate-specific expression pattern of Shh, Shh-Cre activity is also found exclusively in the floor plate [2] (see Figure 1N', N" of Varadarajan et al., 2017 Neuron [3]).

Second, Olig2-Cre is not active in the floor plate. For instance, Varadarajan et al. 2017 Neuron [3] depicted Olig2-Cre active cells and their progeny by crossing Olig2-Cre deleter with Rosa26-gfp reporter mice, and the data show that GFP is not expressed in the floor plate (see Figure 2FF of Varadarajan et al., 2017 Neuron [3]). We have also crossed Olig2-Cre with the reporter allele detecting Cre-mediated recombination and found that Olig2-Cre is not active in the floor plate.

As the reviewer described, Figure 1H of Dessaud et al., 2007 [1] clearly shows that Olig2 is *not* expressed in the MOST medial cells, which are the floor plate. The ventral most domain of the spinal cord in which Olig2 is initially expressed in this paper [1] is the p3 domain, which gives rise to V3 interneurons. Importantly, V3 interneurons do not express Shh.

Taken together, when the patterning of the ventral neural progenitors occurs, Olig2-Cre is *not* active in the floor plate cells, which are the only cell types that express Shh at this stage. Thus, the deletion of the Shh gene by Olig2-Cre should affect neither the expression of Shh in the neural tube nor Shh-dependent patterning of the ventral progenitor domains, including pMN domain.

Finally, the phenotype of Shh-cKO;Olig2-Cre mice further supports the notion that the observed phenotype is *not* caused by Shh deletion in the floor plate. Shh in the floor plate is required for the patterning of the ventral spinal cord, the production of pMN cells, and the generation of motor neurons and ventral interneurons [4]. Thus, if Shh in the floor plate is absent or reduced in Shh-cKO;Olig2-Cre embryos, it would lead to a complete loss or a drastic reduction of Olig2^+^ pMN progenitors, all types of motor neurons, and V2 interneurons. However, we found no significant difference in the number of Olig2^+^ pMN progenitors in the analyzed Shh-cKO;Olig2-Cre and control embryos (Figure 3B,C), indicating that Shh level secreted from the floor plate was adequate to drive the pMN patterning and production. In the revised manuscript, we included the quantification data of the number of Olig2^+^ cells in Shh-cKO;Olig2-Cre and control embryos (Figure 3B,C). We also found that only FoxP1^+^ LMC motor neurons, but neither Lhx3^+^Hb9^+^ MMC, Isl1^+^Hb9^+^ HMC motor neurons nor Lhx3^+^Hb9^-^ V2 interneurons, was reduced in Shh-cKO;Olig2-Cre mice (Figure 3A,C). These data further support the notion that Shh secreted from the floor plate in Shh-cKO;Olig2-Cre mice was adequate to drive the patterning of the ventral spinal cord and the production of pMN cells and that the reduced LMC motor neuron is not a secondary outcome of the defects in neural progenitors.

We hope these address the reviewer’s concern that the observed phenotype can be caused by the deletion of Shh in Olig2^+^ progenitors.

Next, the reviewer #2 stressed to use a motor neuron-specific driver, such as Hb9-Cre line. We have already purchased Hb9-Cre for this purpose, but Hb9-Cre turned out to be problematic for our experiments, because Hb9 is expressed (therefore Hb9-Cre is active) in the notochord, which secretes Shh required for the neural tube development [5]. As Hb9-Cre deletes Shh in the notochord and eliminates Shh required for the ventral neural tube patterning, Hb9-Cre cannot be used for our experiment. To circumvent this issue, we also used Isl1-Cre, whose Cre expression occurs as motor neurons emerge from the progenitors. We generated Shh-cKO with Isl1-Cre and found that Shh-cKO;Isl1-Cre mice have severe deficits in the limb development as Isl1-Cre inactivates Shh in the developing limb [2, 6, 7]. The deficits of limb development in Shh-cKO;Isl1-Cre compounded our analyses of LMC development, given the observed defects of LMC motor neurons can be caused secondarily by the severe developmental defects of the limbs. In summary, Hb9-Cre is inadequate for deleting Shh only in motor neurons, and Olig2-Cre was the best line that we could use to inactive Shh in motor neurons. Finally, our data indicate that the observed phenotypes in Shh-cKO;Olig2-Cre mice are caused by inactivation of Shh in motor neurons, not by the removal of Shh in the floor plate which does not occur in Shh-cKO;Olig2-Cre mice, as described above.

Other concerns include the lack of sufficient analysis to exclude patterning, proliferation or survival defects in the manipulated chick and mouse embryos (reviewer 1 point 1; reviewer 2 points 5 and 6), and the insufficient quantification of the data (reviewer 1 point 2; reviewer 2 points 4, 8, 14, 17, 19, 22-25). We addressed these concerns as explained below. In particular, as for the concern regarding the chick electroporation of Shh knockdown construct, the electroporation efficiency of the floor plate cells is generally very low. In our electroporation scheme, all electroporated cells are labeled by GFP. To better address reviewer's concern, we analyzed the chick embryos that do not express GFP in the floor plate (therefore no inactivation of Shh), which was confirmed by in situ hybridization analyses of Shh expression. We also confirmed that the patterning and cell proliferation are normal in chick embryos analyzed (Figure 2A,C). These rigorous analyses exclude the possibility that any observed phenotypes are caused by the effect of Shh deletion in the progenitors.

With the successful completion of all the requested revisions, we hope that you and the reviewers find this manuscript exciting and convincing. In particular, we hope to emphasize that this story presents at least two highly innovative findings; a novel function for Shh in postmitotic neurons and its critical downstream effector ARHGAP36 in directing the development of the specific type of MNs (LMC-MNs) in the developing spinal cord. Our results identify a new regulatory axis consisting of AKT-ARHGAP36-PKA, which plays crucial roles in Shh signal transduction for LMC specification.

Reviewer #1:

This paper reports a novel role of Shh, produced by postmitotic motor neurons, in the specification of the lateral motor column (LMC) in the chick and mouse spinal cord. The authors show that AKT stabilises the Rho GAP protein ARHGAP36 in the LMC region, and that ARHGAP36 in turn inhibits the activity of PKA and thereby enhances the production of activating forms of Gli proteins.

The identification of a role for Shh in motor neuron specification and the elucidation of the downstream pathway involving a novel regulator and multiple signaling molecules is of significant interest. The data supports the conclusions of the authors and the work is of excellent quality and well presented. I have a few requests to improve the data, the analysis and discussion to strengthen the conclusions of the paper.

Thank you for acknowledging that our findings provide new insights into the role of Shh signaling in motor neuron fate specification through a novel downstream pathway involving multiple signaling molecules. We would also like to thank the reviewer for many insightful suggestions. We are pleased that we were able to address all issues that the reviewer #1 raised, as described below each of the reviewer's point.

1) Shh acts as a mitogen in many systems but the authors rule out that it is the case here by labelling MN progenitors with antibodies to Olig2 and Sox2 in Figure 2C. However the Shh LOF phenotype in Figure 2A only affects the generation of a small minority of MNs, which could be due to a proliferation defect affecting only a small fraction of progenitors which may not be picked up by the current analysis. To rule this out, the authors should examine proliferating cells more directly, eg with a short pulse of EdU. This should detect a putative proliferation defect with more sensitivity than examining total progenitor numbers.

Following the reviewer’s suggestion, we performed a short pulse of BrdU labeling and Ki67 staining. Our analyses revealed that Shh-cKO mice exhibited no significant change in the proliferation of progenitors as monitored by the four independent markers, such as Olig2, Ki67, *Sox2* and BrdU (Figure 3B,C and Figure 3—figure supplement 1B).

2) The ectopic expression of ARHGAP36 in the ventral spinal cord in Figure 5B increases visibly the number of FoxP1+ LMC neurons while Lhx3+ Hb9+ MMCm neurons do not appear affected. However to conclude that ARHGAP36 induces LMC neurons rather than converting MMCm into LMC, requires quantification of the data.

We included the quantification data for FoxP1^+^ LMC and MMC (Lhx3^+^Hb9^+^) neurons (Figure 6B).

3) The conclusion of the experiment in Figure 6A that AKT stabilises ARHGAP36 protein should be strengthened by measuring ARHGAP36 half-life in HEK293T cells in the presence and absence of AKT, and also by showing that manipulating AKT activity does not affect ARHGAP36 transcription.

We included the data showing the half-life of ARHGAP36 protein was prolonged in the presence of AKT when treated with cycloheximide that blocks the protein translation (Figure 7—figure supplement 1).

We also confirmed that AKT inhibitor reduced the protein level of ARHGAP36 by western blotting (Figure 7B, Figure 7—figure supplement 2C) but not the transcription of Arhgap36 by RT-PCR (Figure 7—figure supplement 2D) in mouse embryonic stem cells (mESCs).

4) The regulation of ARHGAP36 expression may need further discussion. Figure 6 shows that AKT controls ARHGAP36 protein stability, but how its transcription is regulated is less clear. Figure 2—figure supplement 1, shows a reduction of its expression in Shh cKO mice. How does Shh regulates its expression and are there other signals involved? Along the same line, the arrow between Shh and P-AKT in Figure 7D may need more explanation.

In Figure 7, to test the effect of AKT activity in the regulation of ARHGAP36 protein stability, we ectopically expressed ARHGAP36 together with different forms of AKT by transfecting HEK293T cells with plasmids that encode ARHGAP36 and AKT genes. As ARHGAP36 is not expressed endogenously in HEK293T cells, in these cells, ARHGAP36 proteins are expressed only from the ARHGAP36-encoding plasmid, in which CMV promoter drives the transcription of ARHGAP36. These experiments show that active AKT increases ARHGAP36 protein levels, suggesting that AKT increases ARHGAP36 proteins likely by stabilizing ARHGAP36 protein, rather than activating the ARHGAP36 promoter transcriptionally, in this experimental setting.

To further test whether endogenous ARHGAP36 transcription is upregulated by AKT in differentiating motor neurons, we used mESC system where ARHGAP36 is induced under motor neuron differentiation condition. In this condition, AKT inhibitor did not affect the mRNA level of ARHGAP36 (see the answer above and Figure 7—figure supplement 2D), suggesting that AKT is not involved in the transcriptional activation of the ARHGAP36 gene.

Then, how does Shh increase ARHGAP36 levels? It is noteworthy that Shh is capable of inducing the activation of phosphoinositide 3-kinase (PI3-kinase)-dependent AKT phosphorylation in cell lines (LIGHT cells and HUBEC cells) [8, 9]. We proposed that Shh expressed in the motor neurons triggers AKT activation, which in turn stabilizes the protein level of ARHGAP36 in motor neurons. Our model predicts that the ARHGAP36 protein level would be decreased in *Shh*-cKO mice.

Reviewer #2:

The manuscript by Nam and cols, entitled "Critical roles of ARHGAP36 as a signal transduction mediator of Shh pathway in lateral motor columnar specification" proposes a novel role for Shh signalling in spinal cord development.

A putative role for Shh activity as a signal instructing the selection of specific MNs subpopulations is novel and attractive. It is based in the observation of Shh expression in postmitotic MNs, which is not fully novel, but has not been well characterized previously.

Authors also find that the Rho GAP family member Arhgap36 is expressed in postmitotic MNs, and propose that it might act as a Shh effector in MN subpopulations selection. In different cell contexts, Arhgap36 has previously been shown to inhibit PKA activity, hence regulating Shh signalling. However, it would be nice to fully define its in vivo relevance, in the context of selection of specific MNs subpopulations. In summary, the manuscript contains some novel and interesting data, however it lacks definitive demonstration of some of the conclusions raised by the authors.

Hence, I believe that, in the present format, the manuscript has important gaps that need to be fulfilled.

We appreciate the acknowledgment that our findings provide a novel role for Shh signaling and its effector ARHGAP36 in generation of specific MNs subpopulation, and we would like to thank the reviewer for many excellent suggestions. As described below, now we provided all quantification, control, and other data, which the reviewer asked to make the manuscript more complete.

Specific points:

In order to propose a new role for Shh signalling as an instructive signal the selection of specific MNs subpopulations, I believe that authors should improve the characterization of the MN subpopulations expressing Shh.

1) In the chick embryo spinal cord, Shh expression has previously been reported in postmitotic motor neurons of the LMC (at stage HH15), extending later to the MMC (Stage HH35) (Figure 1 in Oppenheim et al., 1999). However, Nam and cols, in this manuscript shows Shh expression restricted to LMC neurons at the brachial level. The selected section showed in Figure 1A shows Shh expression restricted to the mLMC (and excluded from lLMC), as well as excluded from MMC. Could authors clarify these contradictory issues?

As the reviewer suggested, we re-examined the expression of Shh more thoroughly with well-defined markers for motor columns in chick embryos (stage HH29) (Figure 1B). Our analyses show that Shh is expressed in LMCm (Isl1^+^/FoxP1^+^) but not LMCl (Isl1^-^/FoxP1^+^) at brachial level, and interestingly, it is expressed in LMCl (Isl1^-^/FoxP1^+^) at lumbar level. Shh expression is clearly excluded from MMC (Lhx3^+^ MNs) at all axial levels, as demonstrated by converging analyses of Lhx3 and Shh (Figure 1B). Overall, our Shh expression analysis results are highly similar to the data in the referenced paper (Oppenheim et al., 1999) [10], despite the minor differences likely due to detection sensitivity variations of Shh antisense probes.

Oppenheim et al. showed nice expression of Shh in chick embryos at different developmental stages. At stage HH15, Shh is expressed in notochord and floor plate but not in postmitotic motor neurons of the LMC (Figure 1A in Oppenheim et al. [10]), which is similar to what we have observed. There is a low level detection of Shh in the ventral horn at stage HH23 (Figure 1C in Oppenheim et al. [10]), which may result from higher level sensitivity of Shh antisense probe. Oppenheim et al. also described that at stage HH35, Shh is expressed in two distinct motoneuron (MN) populations (medial and lateral) and the referee 2 may be referring this medial MNs as MMC. However, this Shh-expressing medial MN population is likely to be LMCm, judging by the cell body location. It is hard to conclude the identity of motor columns without co-expression analyses with motor column markers. Thus, we performed these needed co-expression analyses in Figure 1B.

2) Panel B of this figure (expression in mouse embryos) do not show this restricted expression to mLMC. Are these specie specific differences? Or developmental stage differences?

The expression level of Shh in mouse motor neurons is much lower than that in chick motor neurons. As we examined the comparable developmental stages in mouse and chick embryos, we do not think that the difference in expression levels reflects developmental stage differences. We have observed that overall in situ hybridization analyses tend to work more effectively in chick embryos than in mouse embryos not only for Shh but also for many other genes. This sensitivity differences in in situ hybridization outcomes may reflect species differences, but we are yet to understand what factors exactly contribute to this observation. Nonetheless, our analyses in both mouse and chick embryos demonstrate the expression of Shh in postmitotic motor neurons.

3) A detailed temporo-spatial expression of Shh in postmitotic MNs should be provided. These should be easy to clarify combining with the available and well defined markers for each MN column (see for example Adams et al., 2015)

Following the reviewer’s advice, we re-examined the expression of Shh more thoroughly with well-defined markers for motor columns in chick embryos (stage HH29) (Figure 1B). Our analyses show that Shh is expressed in LMCm (Isl1^+^/FoxP1^+^) but not LMCl (Isl1^-^/FoxP1^+^) at brachial level, and interestingly, it is expressed in LMCl (Isl1^-^/FoxP1^+^) at lumbar level. Shh expression is clearly excluded from MMC (Lhx3^+^ MNs) at all axial levels, as demonstrated by converging analyses of Lhx3 and Shh (Figure 1B).

In order to analyse the role of Shh in MN subtype specification; loss-of-function experiments in chick embryo NT were performed by electroporation at HH stage 13 chick embryos (Materials and methods), at which patterning of the NT is not definitely established (see for example Cayuso et al., 2006).

4) Controls showing quantification of Shh- loss are required, particularly since defects appeared to be mild, despite the length of the experiments (96 hours post-electroporation at HH13). For example, Gli-bs-luc experiments to show the efficiency of the Shh-LOF vector.

In the revised manuscript, we quantified the intensity of Shh ISH signal and confirmed the significant reduction of Shh expression in motor neurons (Figure 2C). Of note, in our electroporation condition, the electroporation efficiency of the floor plate cells is generally very low, likely due to the depth and location of the electrodes. Thus, we expected that Shh knockdown would be mostly effective in motor neurons but not in the floor plate, allowing us to focus on analyzing the effect of Shh-LOF on motor columnar specification without significant changes in progenitor proliferation or early patterning of the neural tube. Our analyses including careful quantification indeed revealed that there’s no significant change in proliferation (BrdU^+^ cells) or ventral neural patterning (Olig2^+^, Nkx2.2^+^ cells in Figure 2A,C). We also found a significant reduction of LMCl motor neurons (Figure 2B,C).

5) Moreover, the role of Shh signalling in proliferation and survival of neural progenitors are also well stablished, hence controls ruling out these defects are required.

In the revised manuscript, we provided the data of BrdU incorporation for proliferation and cleaved caspase 3 staining for apoptotic cells and these results show no significant change in proliferation and survival of neural progenitors (Figure 2A,C).

6) Controls showing markers for progenitors should be included in order to rule out patterning defects.

Now we provided the data that ventral progenitor cells are established properly by staining with Olig2 and Nkx2.2, markers of p2 and p3 domain, respectively, whose expression is dependent on Shh levels in the floor plate (Figure 2A,C).

7) I strongly believe that, in order to analyse a specific role for Shh signalling in postmitotic MN subtype generation, experiments should have been done using a MN specific driver (for example the well characterized HB9) to avoid patterning and proliferation defects.

We have already attempted to use Hb9-Cre for this purpose, but Hb9-Cre turned out to be problematic for our experiments, because Hb9 is expressed (therefore Hb9-Cre is active) in the notochord, which secretes Shh required for the neural tube development [5]. As Hb9-Cre deletes Shh in the notochord and eliminates Shh required for the ventral neural tube patterning, deleting Shh with Hb9-Cre is expected to cause severe patterning and proliferation defects in the neural tube. Thus, Hb9-Cre cannot be used for our experiment. To circumvent this issue, we also used Isl1-Cre, whose Cre expression occurs as motor neurons emerge from the progenitors. We generated Shh-cKO with Isl1-Cre and found that Shh-cKO;Isl1-Cre mice have severe deficits in the limb development as Isl1-Cre inactivates Shh in the developing limb [2, 6, 7]. The deficits of limb development in Shh-cKO;Isl1-Cre compounded our analyses of LMC development, given the observed defects of LMC motor neurons can be caused secondarily by the severe developmental defects of the limbs. In summary, Hb9-Cre is inadequate for deleting Shh only in motor neurons, and Olig2-Cre was the best line that we could use to inactive Shh in motor neurons.

To complement Shh-cKO mouse analysis, we also analyzed chick embryos in which Shh is knocked down in motor neurons, but not in the floor plate (Figure 2A). In our chick electroporation experiments, the electroporation efficiency of the floor plate cells is generally very low and all electroporated cells are labeled by GFP. We analyzed the chick embryos that do not express GFP in the floor plate (therefore no inactivation of Shh), which was further confirmed by in situ hybridization analyses of Shh expression. Our data show that the cell proliferation is normal in chick embryos analyzed. We also examined ventral progenitor markers, such as Nkx2.2 and Olig2, whose expression is dependent on Shh levels in the floor plate to further probe the presence of Shh in the floor plate. These rigorous analyses exclude the possibility that any observed phenotypes are caused by the effect of Shh deletion in the progenitors.

8) Quantitative data indicating the total number of MNs, versus sub-type specific MNs should be provided. It is important to understand whether LMNs have switch their fate, of whether LMNs are not being generated.

Now we provided the quantification data for total number of MNs and sub-type specific MNs in Figure 2C. Knockdown of Shh resulted in reduction of LMCl motor neurons without affecting other motor column subtypes and consequently, the total number of MNs was reduced compared to the uninjected side (Figure 2C). This result indicates that LMC motor neurons are not properly generated or maintained rather than switching to other MN subtypes in the absence of Shh.

9) Quantitative data in Figure 1A panel D shows FoxP1+ cells, are these islet1+? Or islet1-? Same for Hb9, these analyses will discriminate between lLMB vs mLMC.

Quantitative data in Figure 1A panel E shows Lhx3+ cells, same for the combination with additional marker to restrict the phenotype to specific MN subtype

Now we provided the quantification data for each motor column such as LMCl (Hb9^+^/FoxP1^+^ or Isl1^-^/FoxP1^+^), LMCm (Hb9^-^/FoxP1^+^ or Isl1^+^/FoxP1^+^), HMC (Hb9^+^/Isl1^+^) and MMC (Lhx3^+^/Hb9^+^) in Figure 2C.

10) A schematic drawing of the MN columns, including markers and phenotype would be of great help.

We included the schematic drawing of the MN columns with markers and phenotype in Figure 8D.

11) The same holds for the analysis of the Shh-Olig2C mutant phenotype. These experiments should have been done using a MN specific driver (for example the well characterized HB9CRE line, available at Jackson) to avoid patterning and proliferation defects.

We chose Olig2-Cre driver for our conditional knockout experiments because the previous work has shown that Olig2-Cre does not delete the gene in the floor plate cells [1, 11]. Shh in the floor plate is required for the patterning of the ventral spinal cord, the production of pMN cells, and the generation of motor neurons and ventral interneurons [4]. Thus, if Shh in the floor plate is absent or reduced in Shh-cKO;Olig2-Cre embryos, we expect that it would lead to a complete loss or a drastic reduction of Olig2^+^ pMN progenitors, all types of motor neurons, and V2 interneurons. However, there was no significant difference in the number of Olig2^+^ pMN progenitors in the analyzed Shh-cKO;Olig2-Cre and control embryos, suggesting that Shh level secreted from the floor plate was adequate to drive the pMN patterning and production. We included the quantification data of the number of Olig2^+^ cells in Shh-cKO;Olig2-Cre and control embryos (Figure 3C). We also found that only FoxP1^+^ LMC motor neurons including both LMCm and LMCl, but neither MMC (Lhx3^+^Hb9^+^) motor neurons nor HMC (Isl1^+^Hb9^+^) motor neurons, was reduced in Shh-cKO;Olig2-Cre mice (Figure 3C), further indicating that Shh secreted from the floor plate was adequate to drive the patterning of the ventral spinal cord and the production of pMN cells.

Also please see our response to the question 7, regarding why Hb9-Cre can’t be used for Shh-cKO analyses.

12) Please indicate the total number of MNs, versus sub-type specific MNs. Please indicate whether FoxP1+ cells are islet1 and Hb9 +/-.

We included the quantification data for each motor column and total number of MNs in Figure 3C. The number of FoxP1^+^ LMC motor neurons including both LMCm and LMCl was reduced, but neither MMC (Lhx3^+^Hb9^+^) motor neurons nor Isl1^+^Hb9^+^ HMC motor neurons, was changed in Shh-cKO;Olig2-Cre mice (Figure 3C), which results in reduction of total number of MNs in *Shh*-cKO compared to control littermates.

To identify the candidate effector genes, authors searched for target genes of the Isl1-Lhx3 transcriptional complex, well characterized previously by this group. What is the link between this searching strategy and Shh signalling, it is not clear to me, but they identify the Rho GAP family member Arhgap36, which is known to activate Gli transcriptional responses through the inhibition of PKA activities.

While we were analyzing the function of Shh in the columnar specification, ARHGAP36 was reported to activate Gli transcriptional responses through the inhibition of PKA activities. We have examined the expression pattern of putative target genes of the Isl1-Lhx3 complex in the developing spinal cord and found ARHGAP36 as one of the potential MN genes as its expression is very high and specific in MNs.

13) It would be nice to see, among the many genes regulated by the Isl1-Lhx3 transcriptional complex, whether there might be a cluster of HH-signalling components?

We do not see a cluster of HH-signaling components regulated by the Isl1-Lhx3 complex other than ARHGAP36.

*14) Authors identify a HxRE of the Arhgap36 gene, and test the* in vivo *activity by electroporation experiments in chick embryo NT. Please provide quantitative data indicating number of embryos analysed. Provide quantitative data indicating HB9 ectopic cells induced. Please provide electroporation time course, as well as negative control experiments.*

We provided quantitative data for the number of embryos injected, electroporation time course in the figure legend. It is very well established that electroporation of Isl1+Lhx3 induces ectopic Hb9^+^ cells in the dorsal spinal cord [12-14], and we routinely do this experiment in the lab. From this set of experiment, we would like to see the induction of GFP reporter driven by *Arghap36*-enhancer activated by Isl1+Lhx3 in the dorsal spinal cord. Indeed, we could detect very nice GFP expression as well as ectopic Hb9^+^ cells in the dorsal spinal cord by Isl1+Lhx3 expression. We included the counting data of the ectopic Hb9^+^ cells in the dorsal spinal cord (Figure 4F) and negative control experiment with TATA-GFP vector construct that has no HxRE and was not activated by Isl1+Lhx3 (Figure 4E).

15) I believe that the data provided here demonstrate that the Isl1-Lhx3 complex is sufficient to induce Arhgap36 expression, whether this induction is direct is not fully demonstrated.

ChIP assay using mouse spinal cord extracts showed that Isl1 and Lhx3 are strongly recruited to the enhancer region of *Arhgap36* but not to the negative control region of *Untr6* (Figure 4C), indicating that the induction of Arhgap36 by Isl1-Lhx3 complex is direct indeed.

Next authors analyse Arhgap36 expression in postmitotic neurons. This is an important issue, since Shh might be acting either in a paracrine manner, or in a cell autonomous manner, to instruct MNs subtype identity.

16) Please provide a better characterization of Arhgap36 in MN subtypes (co-localization with MN subtype markers) and co-localization (or not) with Shh, with cellular resolution.

We now provided the detailed expression data of *Arhgap36* combining with defined markers for each MN column such as *Arhgap36*/Isl1/FoxP1, *Arhgap36*/Isl1/Hb9, and *Arhgap36*/Lhx3/Hb9 in mouse embryos (Figure 5C). *Arhgap36* is most highly expressed in LMCl (Isl1^-^/FoxP1^+^) region, some in MMC-rhomboideus (Hb9^+^/Lhx3^low^) and a very little in the most medial part of MMC but not in LMCm (Isl1^+^/FoxP1^+^) at cervical level. At thoracic level, *Arhgap36* is also expressed in PGC (FoxP1^+^/Isl1^+^) and HMC (Isl1^+^/Hb9^+^) MNs, although the expression level is relatively lower. At lumbar level, *Arhgap36* is relatively highly enriched in LMCl (Isl1^-^/FoxP1^+^) and show very low level in the most medial part of MMC. To examine the co-localization of *Arhgap36* with Shh, we performed in situ hybridization of Shh and IHC of *Arhgap36* in mouse E12.5 spinal cord at cervical level. Shh is co-localized with *Arhgap36* mostly in LMCl region (Figure 5D).

In summary, mouse Shh is mainly expressed in LMCl of the mouse spinal cord that is co-localized with *Arhgap36*, while chick Shh is expressed in LMCm at cervical level and LMCl at lumbar level of chick spinal cord where chick *Arhgap36* is broadly expressed within the spinal cord. Although there is a discrepancy in the expression pattern of Shh in mouse and chick spinal cord, *Arhgap36* seems to be coexpressed with Shh in both species and the regulatory axis of Shh-ARHGAP36 can still function in both mouse and chick spinal cord.

17) Please provide Arhgap36 expression data in chick embryos MNs. It would be interesting to see the conservation of it expression in MNs.

In situ hybridization of chick *Arhgap36* showed rather broad but specific and high expression within the spinal cord compared to other tissues in the chick embryo (Figure 7—figure supplement 3).

Next authors test whether the Shh-pathway is activated upon over-expression of Arhgap36. Experiments in chick embryo NT were performed by electroporation of ARHGAP36 at HH stage 13 chick embryos (Materials and methods). These gain-of-function experiments produce NT overgrowth, corresponding to the over-activation of Shh signalling, in addition to ventralization of the entire NT. Quantitative data indicating number of embryos analysed is missing, electroporation time course, as well as quantification of marker-specific ectopic cells should be provided.

We provided quantitative data for the number of embryos injected, electroporation time course in the figure legend and included the quantification data for FoxP1^+^ LMC and MMC (Lhx3^+^/Hb9^+^) neurons (Figure 6B).

*18) I believe that these experiments are controls of the capacity of ARHGAP36 in activating Shh-mediated responses* in vivo*. Since this has been previously documented in other systems, these experiments can go as supplementary information.*

We moved these data to supplementary information (Figure 6—figure supplement 1).

19) Authors claim that over-expression of ARHGAP36 in ventral NT, harvested at 4 dpe the number of FoxP1+ LMC neurons increased drastically, suggest that ARHGAP36 is sufficient to direct LMC fate-determination. Again here, I believe that these experiments should have been done using a MN HB9 specific driver to avoid patterning and proliferation defects. With the present experimental design, it is very difficult to assign a direct role for ARHGAP36 in postmitotic MN subpopulations selection, other that the role in MN progenitors

We used Gal4/UAS system to drive the motor neuron specific expression of *Arhgap36*. We generated Hb9-Gal4 construct where Gal4 DNA binding transactivation domain is expressed driven by motor neuron specific Hb9 promoter and UAS-*Arhgap36* construct where *Arhgap36* is expressed through the binding of Gal4 to the UAS response element. Using this system, we could direct the expression of *Arhgap36* into postmitotic MNs and this experiment also resulted in the specific increase of FoxP1^+^ LMC neurons but had no effect on MMC (Lhx3^+^/Hb9^+^) neurons (Figure 6).

Again in these experiments, quantitative data indicating number of embryos analysed is required, electroporation time course is missing. Quantification of marker-specific ectopic cells should be provided.

We now provided the quantitative data of number of embryos in the figure legend and quantification data for marker^+^ cell numbers in Figure 6.

Next, authors show that PKA activity is inhibited by ARHGAP36. However, this observation is not novel, since Eccles et al. (2016) showed that ARHGAP36 combines two distinct inhibitory mechanisms to antagonise PKA signalling; it blocks PKA catalytic activity and it targets PKAc for ubiquitin-mediated degradation, resulting in activation of the Shh pathway.

20) I don't see the point in analysing other targets but PKA in the context of MN specification, hence I believe that panels C,D,E from Figure 5 are not relevant in this context.

We moved these data to supplementary figures (Figure 6—figure supplement 1).

21) Again, in the context of Shh/ ARHGAP36 signalling in MN subtype selection, I do not see the relevance of the AKT experiments shown if Figure 6. I believe that should be removed.

Moreover, Zhang et al. (2019) find that Patched1 interacts with ArhGAP36 to the centrosome and stabilizes the PKA negative regulator (this paper should be quoted in the manuscript).

Zhang et al., Patched1-ArhGAP36-PKA-Inversin axis determines the ciliary translocation of Smoothened for Sonic Hedgehog pathway activation. Proc Natl Acad Sci U S A. 2019 Jan 15;116(3):874-879

We cited this paper in the introduction. As Shh pathway plays critical roles in embryonic development, tissue homeostasis and tumorigenesis, it is important to define the regulatory network modulating multiple components involved in the pathway. We found that AKT stabilizes the Arhgpa36 protein level and further potentiates the Shh activity in MN subtype generation, which is quite novel and this regulatory axis can be broadly implicated in various Shh-dependent conditions.

22) Authors show reduced expression of ARHGAP36 in Shh-KO mice (Figure S4), again without any quantitative data.

We provided the quantitative data (Figure 3—figure supplement 1).

Finally, authors generate an ARHGAP36 mutant mice. Authors claim that at early developmental stages, there was no defect in MN generation. Quantitative data on this control observation should be provided.

We included the data showing that there’s no defect in proliferation (BrdU), ventral neural patterning (Nkx2.2, Olig2) and overall MN generation (Hb9) at E11.5 and provided quantification data in Figure 8—figure supplement 1.

23) Analysis of the phenotype generated by ARHGAP36_CRISPR mutant in MNs at the brachial and the thoracic levels of the developing spinal cord, again require quantitative data indicating that the total number of MNs, have not changed at thoracic levels.

We included the quantification data of HMC (Hb9^+^/Isl1^+^), MMC (Lhx3^+^/Hb9^+^), PGC (nNOS^+^) and total number of MNs at thoracic levels (Figure 8C).

24) Quantitative data indicating that the total number of MMC and the total number of V2 interneurons, have not changed at brachial levels.

We included the quantification data of MMC (Lhx3^+^/Hb9^+^) and V2-INs (Chx10^+^/Lhx3^+^) at brachial levels (Figure 8C).

25) Quantitative data indicating the total MN numbers versus sub-type specific MNs should be provided. It is important to understand whether, in the absence of ARHGAP36, LMNs have switch their fate, of whether LMNs are not being generated.

We included the quantification data of LMCm (Isl1^+^/FoxP1^+^), LMCl (Hb9^+^/FoxP1^+^, Lhx1^+^/FoxP1^+^), HMC (Hb9^+^/Isl1^+^), MMC (Lhx3^+^/Hb9^+^) at the brachial and thoracic levels (Figure 8C). In *Arhgap36* KO mice, FoxP1^+^ LMC including LMCm and LMCl neurons reduced, while there is no compensatory increase in non-LMC type motor neurons that we examined, and then as a result this leads to the reduction of total MNs (Figure 8C). The increase of active Caspase3^+^ cells in the motor neuron area of *Arhgap36* KO mice (Figure 8—figure supplement 1) suggests that prospective LMC neurons undergo apoptotic cell death in the absence of *Arhgap36*. It remains unclear, however, if cell death is a direct outcome of loss of *Arhgap36* in promoting cell survival or an indirect outcome of the failure of acquiring or maintaining the correct LMC identity. Importantly, previous studies show that postmitotic motor neuron fate specification and their survival are closely linked and it is difficult to distinguish the phenotypes between the fate specification and survival of postmitotic motor neurons. After cell cycle exit, the 'generic' motor neurons diversify into distinct motor neuron subtypes, including LMC. Retinoic acid (RA) signaling plays a role in the specification and maintenance of forelimb LMC identity [15-21]. The loss of RA from motor neurons leads to a reduced number of LMC neurons without a significant change in the number of MMC neurons [21], similar to our *Arhgap36* KO embryos. The ectopic activation of RA signaling in thoracic motor neurons impairs non-LMC type motor neuron differentiation and ultimately triggers apoptotic cell death [19]. One potential mechanism that links the fate specification and cell survival is that motor neurons which did not acquire the correct cell identity fail to receive target-derived neurotrophic signals.

References:

1) Dessaud, E., et al., Interpretation of the sonic hedgehog morphogen gradient by a temporal adaptation mechanism. Nature, 2007. 450(7170): p. 717-20.

2) Harfe, B.D., et al., Evidence for an expansion-based temporal Shh gradient in specifying vertebrate digit identities. Cell, 2004. 118(4): p. 517-28.

3) Varadarajan, S.G., et al., Netrin1 Produced by Neural Progenitors, Not Floor Plate Cells, Is Required for Axon Guidance in the Spinal Cord. Neuron, 2017. 94(4): p. 790-799.e3.

4) Jessell, T.M., Neuronal specification in the spinal cord: inductive signals and transcriptional codes. Nat Rev Genet, 2000. 1(1): p. 20-9.

5) Harrison, K.A., et al., Pancreas dorsal lobe agenesis and abnormal islets of Langerhans in Hlxb9-deficient mice. Nat Genet, 1999. 23(1): p. 71-5.

6) Yang, L., et al., Isl1Cre reveals a common Bmp pathway in heart and limb development. Development, 2006. 133(8): p. 1575-85.

7) Itou, J., et al., Islet1 regulates establishment of the posterior hindlimb field upstream of the Hand2-Shh morphoregulatory gene network in mouse embryos. Development, 2012. 139(9): p. 1620-9.

8) Kanda, S., et al., Sonic hedgehog induces capillary morphogenesis by endothelial cells through phosphoinositide 3-kinase. J Biol Chem, 2003. 278(10): p. 8244-9.

9) Riobo, N.A., et al., Phosphoinositide 3-kinase and Akt are essential for Sonic Hedgehog signaling. Proc Natl Acad Sci U S A, 2006. 103(12): p. 4505-10.

10) Oppenheim, R.W., et al., Modulation of early but not later stages of programmed cell death in embryonic avian spinal cord by sonic hedgehog. Mol Cell Neurosci, 1999. 13(5): p. 348-61.

11) Sagner, A., et al., Olig2 and Hes regulatory dynamics during motor neuron differentiation revealed by single cell transcriptomics. PLoS Biol, 2018. 16(2): p. e2003127.

12) Thaler, J.P., et al., LIM factor Lhx3 contributes to the specification of motor neuron and interneuron identity through cell-type-specific protein-protein interactions. Cell, 2002. 110(2): p. 237-49.

13) Lee, S., et al., STAT3 promotes motor neuron differentiation by collaborating with motor neuron-specific LIM complex. Proc Natl Acad Sci U S A, 2013. 110(28): p. 11445-50.

14) Cho, H.H., et al., Isl1 directly controls a cholinergic neuronal identity in the developing forebrain and spinal cord by forming cell type-specific complexes. PLoS Genet, 2014. 10(4): p. e1004280.

15) Sockanathan, S. and T.M. Jessell, Motor neuron-derived retinoid signaling specifies the subtype identity of spinal motor neurons. Cell, 1998. 94(4): p. 503-14.

16) Solomin, L., et al., Retinoid-X receptor signalling in the developing spinal cord. Nature, 1998. 395(6700): p. 398-402.

17) Diez del Corral, R., et al., Opposing FGF and retinoid pathways control ventral neural pattern, neuronal differentiation, and segmentation during body axis extension. Neuron, 2003. 40(1): p. 65-79.

18) Novitch, B.G., et al., A requirement for retinoic acid-mediated transcriptional activation in ventral neural patterning and motor neuron specification. Neuron, 2003. 40(1): p. 81-95.

19) Sockanathan, S., T. Perlmann, and T.M. Jessell, Retinoid receptor signaling in postmitotic motor neurons regulates rostrocaudal positional identity and axonal projection pattern. Neuron, 2003. 40(1): p. 97-111.

20) Vermot, J., et al., Retinaldehyde dehydrogenase 2 and Hoxc8 are required in the murine brachial spinal cord for the specification of Lim1+ motoneurons and the correct distribution of Islet1+ motoneurons. Development, 2005. 132(7): p. 1611-21.

21) Ji, S.J., et al., Mesodermal and neuronal retinoids regulate the induction and maintenance of limb innervating spinal motor neurons. Dev Biol, 2006. 297(1): p. 249-61.
